# Including the urbanization gradient in people‐centered wildlife conservation in Amazonia

**DOI:** 10.1111/cobi.70049

**Published:** 2025-04-28

**Authors:** Lísley P. Lemos, Denis Valle, Thaís Queiroz Morcatty, Willandia Chaves

**Affiliations:** ^1^ Department of Fish and Wildlife Conservation Virginia Polytechnic Institute and State University (Virginia Tech) Blacksburg Virginia USA; ^2^ Rede de Pesquisa para Estudos sobre Diversidade Conservação e Uso da Fauna na Amazônia (RedeFauna) Manaus Brazil; ^3^ Mamirauá Sustainable Development Institute Tefé Brazil; ^4^ Center for Biology and Society Arizona State University Tempe Arizona USA; ^5^ School of Forest, Fisheries, and Geomatics Sciences University of Florida Gainesville Florida USA; ^6^ Department of Geography University College London London UK

**Keywords:** indirect questions, multisited households, peri‐urban, policy, rural, wild meat, wildlife use, carne silvestre, hogares multisede, periurbano, políticas, preguntas indirectas, rural, uso de fauna, 间接问题, 多住址家庭, 城市周边, 农村, 野味, 野生动物利用, 政策

## Abstract

Conservation policy in the Amazon traditionally focuses on rural areas, overlooking the socioecological roles of urban populations. This oversight can hinder sustainability by neglecting rural–urban connections. We compared the prevalence and quantity of wild meat consumed, bartered, and traded commercially in rural, peri‐urban, and urban areas of the Brazilian Amazon to inform policies aimed at including local people in conservation. We also examined social factors influencing wildlife access. These factors included household management (single vs. dual adult households), household dependency (ratio of minors to working adults), residence status (single‐sited vs. multisited households), frequency of rural area visits by urban residents, and market access by peri‐urban and rural residents. We surveyed 782 households in Manaus and Carauari (Manaus: 299 urban, 90 peri‐urban, 120 rural; Carauari: 159 urban, 41 peri‐urban, 73 rural) about social factors related to wildlife used that are linked to urbanization. Results revealed widespread wildlife use across urbanization categories. The percentage of urban households that consumed (Manaus 22%, Carauari 57%), bartered (Manaus 17%, Carauari 30%), and traded (Manaus 21%, Carauari 7%) wildlife was substantial. Market access was higher in Manaus than in Carauari. Commercial trade in chelonians and barter of mammals and birds increased as access to markets increased. Commercial wildlife trade was present in urban households (Carauari 21% [95% CI 7–34], Manaus 16% [95% CI 6–26]) but higher in peri‐urban Manaus (chelonian trade 44% [95% CI 22–62]). Given these high prevalence levels, especially near expanding urban areas, such as Manaus, wildlife barter and commercial trade likely contribute to unsustainable harvesting pressures, affecting people's sovereignty. Our research underscores the need for inclusive policies that regulate subsistence hunting to uphold rural rights; integration of fish and wildlife management in community‐based conservation frameworks to enhance food security and reduce wildlife dependence; and inclusion of wildlife users in integrated development programs through community‐based conservation to curtail wildlife trade, ultimately creating sustainable and just pathways for the urbanizing Amazonia.

## Abstract (es)

Inclusión del gradiente de urbanización en la conservación de fauna enfocada en personas en la Amazonia

**Resumen:**

La política de conservación en la Amazonia se centra tradicionalmente en las zonas rurales, ignorando las funciones socioecológicas de las poblaciones urbanas. Este descuido puede obstaculizar la sostenibilidad al descuidar las conexiones entre las zonas rurales y urbanas. Comparamos la prevalencia y la cantidad de carne silvestre consumida, intercambiada y comercializada en zonas rurales, periurbanas y urbanas de la Amazonia brasileña con el fin de fundamentar las políticas destinadas a incluir a la población local en la conservación. También analizamos los factores sociales que influyen en el acceso a la fauna. Estos factores incluían la gestión de los hogares (hogares unipersonales frente a hogares con dos adultos), la dependencia de los hogares (proporción de menores frente a adultos que trabajan), el estatus de residencia (hogares unipersonales frente a hogares multisede), la frecuencia de las visitas a las zonas rurales por parte de los residentes urbanos y el acceso al mercado por parte de los residentes periurbanos y rurales. Encuestamos a 782 hogares de Manaos y Carauari (Manaos: 299 urbanos, 90 periurbanos, 120 rurales; Carauari: 159 urbanos, 41 periurbanos, 73 rurales) sobre factores sociales relacionados con el uso de la fauna vinculados a la urbanización. Los resultados revelaron un uso generalizado de la fauna silvestre en todas las categorías de urbanización. El porcentaje de hogares urbanos que consumían (Manaos, 22%; Carauari, 57%), intercambiaban (Manaos, 17%; Carauari, 30%) y comerciaban (Manaos, 21%; Carauari, 7%) con animales silvestres fue considerable. El acceso al mercado fue mayor en Manaos que en Carauari. El comercio de quelonios y el trueque de mamíferos y aves aumentaron a medida que aumentaba el acceso a los mercados. El comercio de fauna silvestre estaba presente en los hogares urbanos (Carauari 21% [IC 95%: 7‐34], Manaus 16% [IC 95%: 6‐26]), pero era mayor en la zona periurbana de Manaus (comercio de quelonios 44% [IC 95%: 22‐62]). Dados estos altos niveles de prevalencia, especialmente cerca de zonas urbanas en expansión, como Manaos, es probable que el trueque y el comercio de especies silvestres contribuyan a presiones de recolección insostenibles, afectando a la soberanía de las personas. Nuestra investigación subraya la necesidad de políticas inclusivas que regulen la caza de subsistencia para defender los derechos rurales; la integración de la gestión de la pesca y la vida silvestre en marcos de conservación comunitarios para mejorar la seguridad alimentaria y reducir la dependencia de la vida silvestre; y la inclusión de los usuarios de la vida silvestre en programas de desarrollo integrados a través de la conservación comunitaria para reducir el comercio de vida silvestre, creando en última instancia vías sostenibles y justas para la Amazonia en proceso de urbanización.

## 将城市化梯度纳入亚马逊地区以人为中心的野生动物保护工作

**【摘要】** 传统的亚马逊地区的保护政策侧重于农村地区, 而忽视了城市人口在社会生态方面的影响。这可能会因忽视城乡联系而阻碍可持续发展。本研究比较了巴西亚马逊地区农村、城市周边和城市地区的野味食用、物物交换和商业交易的普遍程度和数量, 以指导旨在推动当地社区参与的保护政策。我们还分析了人们是否获取野生动物的社会影响因素, 包括家庭管理(单亲家庭与双亲家庭)、家庭依赖性(未成年人与工作成年人的比例)、居住状况(单住址家庭与多住址家庭)、城市居民访问农村地区的频率, 以及城市周边和农村居民访问集市的情况。研究共调查了马瑙斯和卡劳阿里的782个家庭(马瑙斯:299个城市家庭, 90个城市周边家庭, 120个农村家庭;卡劳阿里:159个城市家庭, 41个城市周边家庭, 73个农村家庭), 以分析与城市化相关的野生动物利用的社会因素。结果显示, 野生动物利用在不同城市化类型的家庭中都十分普遍。食用(马瑙斯22%, 卡劳阿里57%)、物物交换(马瑙斯17%, 卡劳阿里30%)和交易(马瑙斯21%, 卡劳阿里7%)野生动物的城市家庭占很大比例。在马瑙斯访问集市的机会多于卡劳阿里。随着访问集市的机会增加, 龟鳖类的商业贸易以及哺乳动物和鸟类的物物交换也在增加。城市家庭存在野生动物商业贸易的行为(卡劳阿里21%, 95%置信区间为7–34%;马瑙斯16%, 95%置信区间为6–26%), 但在马瑙斯周边更高(龟鳖类贸易占44%, 95%置信区间为22–62%)。考虑到这些活动如此流行, 尤其是在马瑙斯等不断扩大的城市地区附近, 野生动物的物物交换和商业贸易很可能会产生不可持续的捕猎压力, 影响人们的主权。我们的研究强调, 需要制定包容性政策, 规范化生计狩猎, 以维护农村地区的权利;将鱼类和野生动物管理纳入基于社区的保护框架, 以加强粮食安全, 减少对野生动物的依赖;通过基于社区的保护, 将野生动物使用者纳入综合发展计划, 以遏制野生动物交易, 最终为亚马逊地区的城市化开辟可持续发展的公正道路。**【翻译:胡怡思;审校:聂永刚】**

## INTRODUCTION

Contemporary conservation efforts prioritize expanding local initiatives to curb the effects of rapid human growth, deleterious industries, and large‐scale degradation (IPBES, 2019). In Amazonia, pathways to upscale conservation initiatives to promote positive outcomes for biodiversity and human populations include being locally grounded, encompassing sectorial diversity, and promoting interactions across landscapes (Londres et al., 2023). Thus, conservation strategies that include interconnectedness between rural and urban areas are crucial to addressing global environmental change (Elmqvist et al., 2021).

Urbanization in the Brazilian Amazon is characterized by the interconnectedness of rural and urban systems, but unfortunately, integrated environmental policies are lacking (Pinho et al., 2014). Frequently, urban areas are referred to as “urbanized forest” (Becker, 2005) or “rural cities” (Padoch et al., 2008), emphasizing rural economies in urban centers. Throughout Amazonia, rural‐to‐urban migration and peri‐urbanization—the absorption of rural areas into city regions (Phadke, 2020)—have blurred the rural–urban divide. This continual transition challenges biodiversity conservation. A better grasp of how urbanization affects biodiversity use is critical to addressing threats and delineating adequate policies.

Blurred rural–urban boundaries in Amazonia offer insight into diverse wildlife uses. Urban hunters illustrate this diversity in their strategies, such as adapting transportation systems to access peripheric hunting areas, changing communication to circumvent enforcement (Van Vliet et al., 2015), and reusing abandoned rural areas to hunt (Parry et al., 2009). Wild meat consumption, though traditionally considered rural, occurs in Amazonian cities and feeds clandestine economies (El Bizri et al., 2020). Staggeringly, around 1.7 million turtles and tortoises were estimated to be consumed annually in urban areas of Amazonas state alone (Chaves, et al., 2021), but most conservation initiatives focus on rural consumers. This indicates that although growing urban demand may affect rural sovereignty, addressing wildlife‐related issues across urbanization gradients emerges as a pathway for collaborative policies (Ingram et al., 2021). Wild meat reliance in rural Amazonian households is among the highest in the Global South, both for consumption and income (Nielsen et al., 2018). Yet, rural–urban migration also facilitates urban access, linking gradient extremes. With high internal migration, nearly two thirds of the Brazilian Amazon's population lived in urban areas in the 2010s (IBGE, 2022). New rural–urban migrants tend to consume more wildlife (Torres et al., 2022). People who use resources from and work in both rural and urban areas further promote rural–urban flows and demand for wild foods through their activities in both settings (Padoch et al., 2008).

**FIGURE 1:**
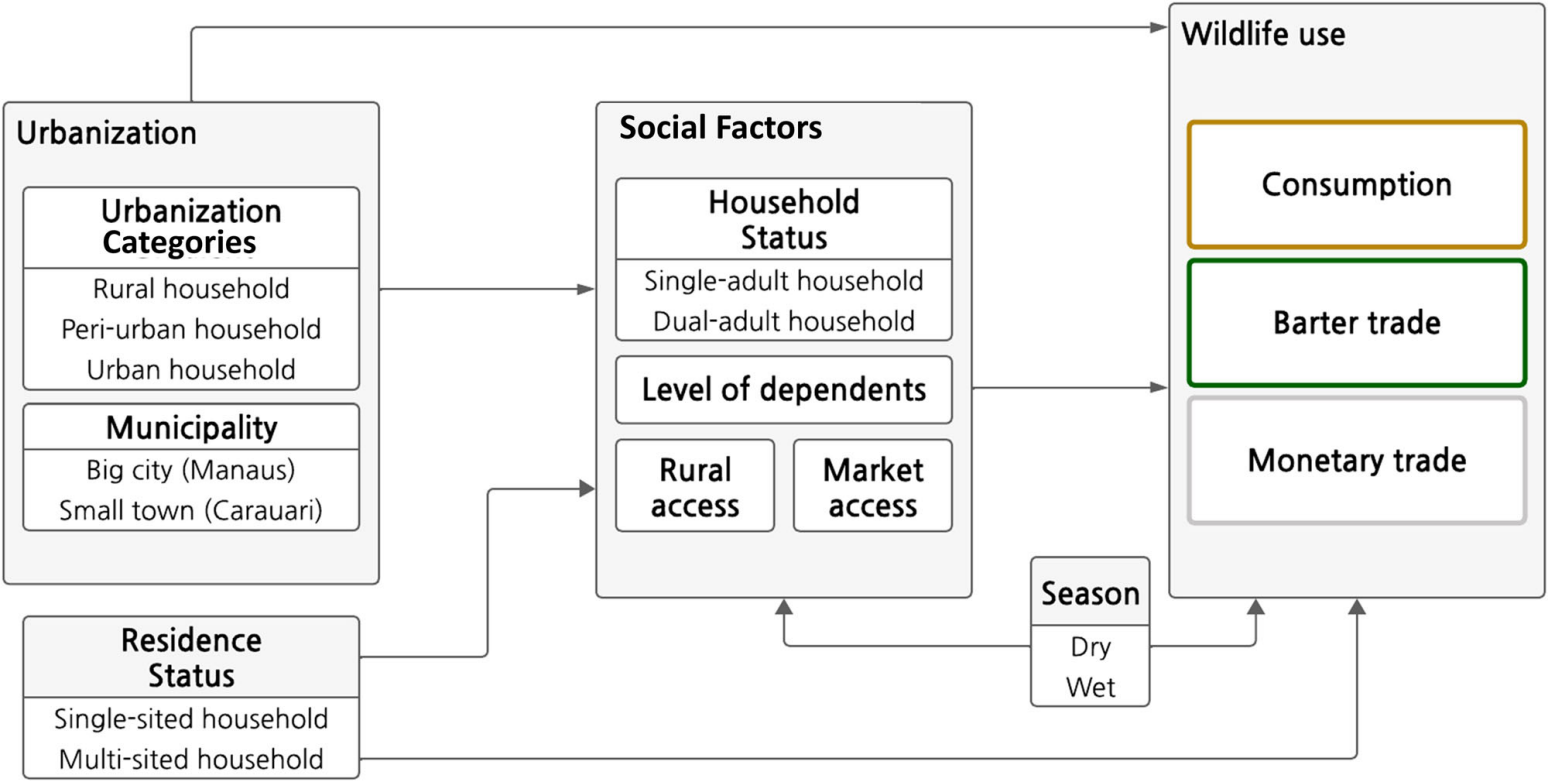
Conceptual framework of factors influencing access to wildlife and household wildlife use across rural, peri‐urban, and urban areas in the Brazilian Amazon. Residence status refers to whether people used natural resources and worked near the site of the residence (single‐sited households) or used both urban and non‐urban areas (multisited households).

We surveyed adult wildlife users in rural, peri‐urban, and urban areas of Manaus and Carauari on the prevalence and quantity of wild meat (meat from wild animals) they used to examine social factors affecting wildlife access. We define access to wildlife as people's ability to obtain and benefit from using meat from turtles, tortoises (hereafter chelonians), mammals, and birds (Ribot & Peluso, 2003). We aimed to determine how social factors affect rural, peri‐urban, and urban use of wild meat to inform inclusive, people‐centered conservation. We considered urbanization a multidimensional process characterized by urban growth primarily driven by rural‐to‐urban migration (Browder & Godfrey, 1997) and assessed it based on categories (rural, peri‐urban, urban) and municipality size (small town, large city) (Figure 1). We also considered the effect of residence status (defined in “METHODS”) on social factors affecting wildlife use (Figure 1). We investigated the effect of urbanization on social factors and examined how these factors influence wildlife use across rural, peri‐urban, and urban areas (Figure 1).

## METHODS

### Study sites

**FIGURE 2:**
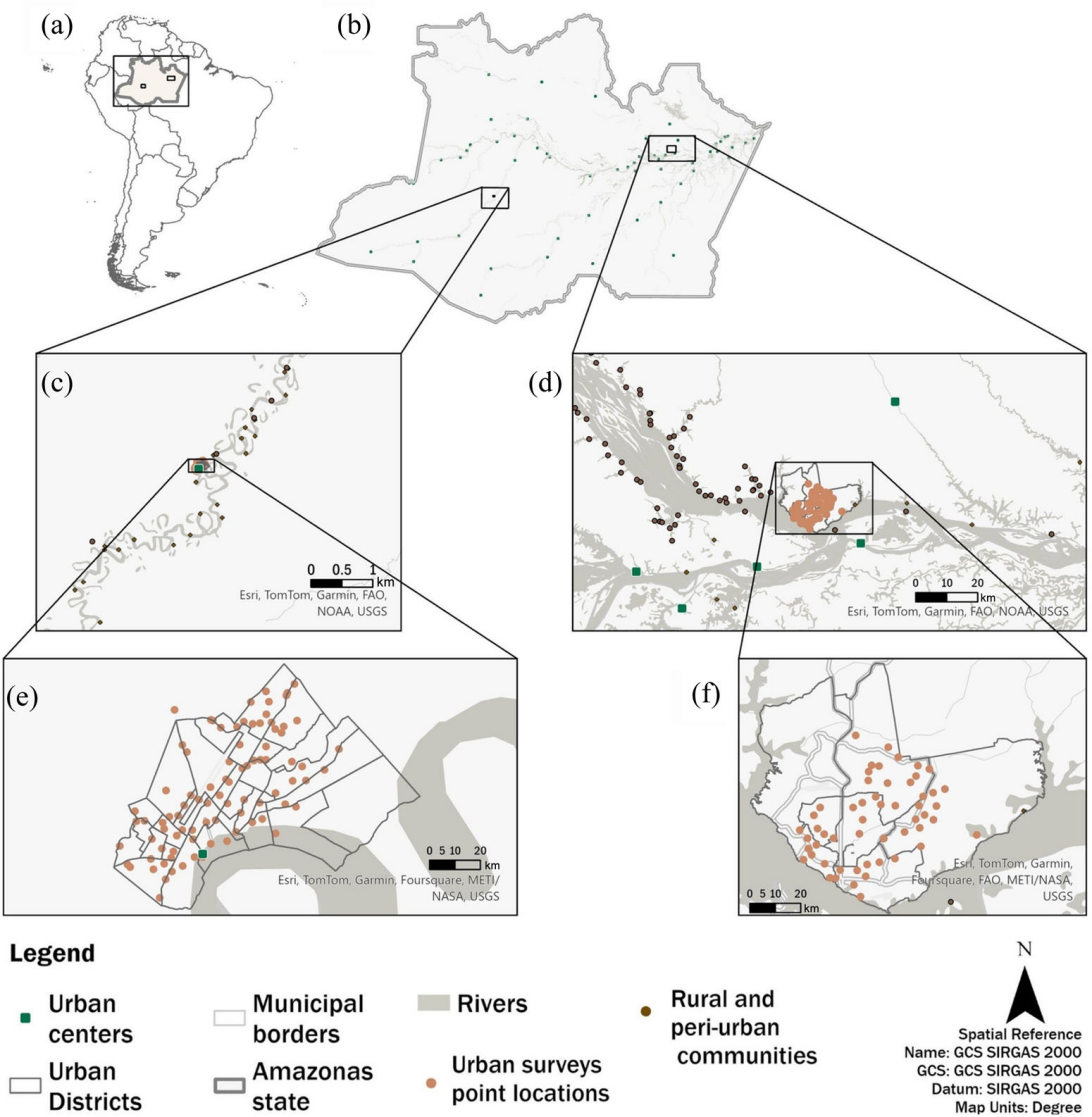
Sites and the urbanization gradient in Manaus and Carauari where people were surveyed relative to (a) studied municipalities, Amazonas state, and South American countries; (b) municipalities and areas sampled in Amazonas state; (c) surveyed rural, peri‐urban, and urban areas in Carauari; (d) surveyed rural, peri‐urban, and urban areas in Manaus; (e) urban districts and random survey locations in Carauari; and (f) urban districts and random survey locations in Manaus.

We used a comparative design to assess how urbanization affects the prevalence and quantity of meat from wild animals consumed, bartered, and commercially traded in rural, peri‐urban, and urban areas. We surveyed households on their wildlife use in Manaus, the largest city in the Amazon basin, and Carauari, a remote municipality in Amazonas state (Figure 2). These sites were selected to represent extremes of urbanization levels in the Brazilian Amazon based on urban area (Manaus 487 km^2^, Carauari 10 km^2^) and population size (Manaus 2,063,689 inhabitants, Carauari 28,742 inhabitants) (IBGE, 2022).

We defined the urban area as the urbanized portion within municipal boundaries (IBGE, 2010), peri‐urban area as the area ranging from the border of the urban area up to 40 km out (or 12 h by boat during the wet season), and rural area as anywhere beyond the peri‐urban border (Hutchings et al., 2022). We used these categories as proxies for a rural–urban gradient (Appendix S4) but recognize this is a spatial simplification of the complex nature of urbanization and peri‐urbanization processes (Gonçalves et al., 2017).

### Household surveys

We conducted our surveys from May to August 2022. Our study design was approved by the Institutional Review Board (protocol 1040) and by the Comitê de Ética em Pesquisa (CAAE 6.0000.8117). We also obtained consent from local organizations and research participants prior to administering the surveys.

People are likely to underreport wildlife use due to its illegality in Brazil (Brasil, 1967, 1998). To obtain more honest answers on wildlife use, we used a randomized response technique known as an unrelated question design (Blair et al., 2015). This indirect technique anonymizes the responses of research participants, decreasing bias in reported wild meat use (Chaves, et al., 2021).

In each sampled household, we asked participants questions that could be related to either illegal (sensitive) or legally obtained (nonsensitive) items. We used dominoes as randomizers. One domino (out of 6) was chosen randomly from a cloth bag by participants. Four dominoes had 2 dots. Participants who drew 2 dots answered questions about sensitive items (wild meat). Two dominoes had one dot. Participants who drew one dot answered questions about nonsensitive items (legal food items and merchandise). Participants answered general questions (e.g., “How many units of this item did you consume in the dry/wet season?”) without explicitly naming the item, ensuring the interviewer did not know the outcome of the randomization (Appendix S1). Survey questions were tested before being applied and are available in Appendix S2.

We assessed the following social factors potentially affected by urbanization and that may influence wildlife use: whether the household was managed by one adult or 2 adults and the level of dependency in the household (i.e., number of people <18 years old divided by the number of working people ≥18 years old). We also assessed access to natural resources as the number of visits to a rural area by any urban household member in the wet season. Similarly, we assessed access to urban markets as of visits by any peri‐urban or rural household member to the closest urban center in the wet season. We assessed household residence status as multisited or single sited. Single‐sited households used natural resources and worked near the site of the residence. People from multisited households used both urban and rural areas.

### Sampling design

We conducted 782 household surveys, 509 in Manaus (120 rural, 90 peri‐urban, 299 urban area) and 273 in Carauari (73 rural, 41 peri‐urban, 159 urban area). We randomly selected households in each area (sampling design in Appendix S1). Adults aged 18–85 years provided household information and chose who answered the survey.

### Data analyses

We used a negative binomial regression to assess the effect of municipality, urbanization categories, and residence status on the frequency of visits to the urban center by rural and peri‐urban households and of visits to rural and peri‐urban areas by urban households. We used the dplyr package (Wickham et al., 2021) and fitted models with the glmmTMB package (Brooks et al., 2017).

We used a logistic regression to assess the effect of urbanization on the prevalence of single‐ or dual‐adult households, using the same set of predictors described above. We used a logistic regression to assess the effect of municipality, urbanization categories, and residence status on the prevalence of dependents over working‐age adults.

We used customized Bayesian models to estimate the prevalence of consumption, barter, and commercial trade of wild mammals, birds, and chelonians by household in Manaus and Carauari for each urbanization category during the dry and wet seasons. To do this, we used the JAGS modeling platform (Plummer, 2003) with 25,000 samples from the posterior distribution. We discarded the first 50,000 iterations from the burn‐in period. We focused on wildlife use (binary variable) and assumed a Bernoulli distribution (Chaves, et al., 2021). We also estimated the quantity used (kilograms or units used per month) by households with ordered logistic regression (statistical model in Appendix S1).

We used ordered logistic regression models to look at additional factors associated with the consumption, barter, and commercial trade of wild meat (see Appendix S1 and Table 1 for details). Our first model included spatial factors, and the second model included social factors associated with wildlife use. We ran separate models for rural access and market access because each included a different set of households. Season (dry and wet) was included in all models. All analyses were conducted in RStudio (R Core Team, 2020).

**TABLE 1:** Models and variables used to assess the effects of urbanization and social factors on wildlife use across rural, peri‐urban, and urban areas in the Brazilian Amazon.

Model	Type of analysis	Response variable	Predictor variable
Spatial factors associated with access to urban markets by rural and peri‐urban households	Negative binomial regression	Number of visits to the urban center	Municipality (Manaus = 1, Carauari = 0) Residence status (multisited = 1, single‐sited = 0) Urbanization gradient (peri‐urban = 1, urban = 0)
Spatial factors associated with access to rural resources by urban households	Negative binomial regression	Number of visits to the rural area	Municipality (Manaus = 1, Carauari = 0) Residence status (multisited = 1, single‐sited = 0)
Spatial factors associated with social factors	Logistic regression	Prevalence of dual‐adult households	Municipality (Manaus = 1, Carauari = 0) Residence status (multisited = 1, single‐sited = 0) Urbanization gradient (peri‐urban = 1, urban = 0 and rural = 1, urban = 0)
	Logistic regression	Prevalence of dependents (<18 years old) over working‐age adults (≥18 years old)^a^	Municipality (Manaus = 1, Carauari = 0) Residence status (multisited = 1, single‐sited = 0) Urbanization gradient (peri‐urban = 1, urban = 0 and rural = 1, urban = 0)
Spatial factors associated with wildlife consumption, barter or commercial trade	Ordinal logistic regression	Kilograms of mammal and bird meat used per month or units of turtles and tortoises used per month	Municipality (Manaus = 1, Carauari = 0) Residence status (multisited = 1, single‐sited = 0) Urbanization gradient (peri‐urban = 1, urban = 0 and rural = 1, urban = 0) Season (dry season = 1, wet season = 0)
Visits to the rural area and wildlife consumption, barter or commercial trade			Rural access (number of visits to the rural or peri‐urban areas in the last season) Season (dry season = 1, wet season = 0)
Visits to the urban area and wildlife consumption, barter or commercial trade			Rural access (number of visits to the urban area in the last season) Season (dry season = 1, wet season = 0)
Social factors associated with wildlife consumption, barter or commercial trade			Household status (dual‐adult = 1, single‐adult = 0) Number of dependents Season (dry season = 1, wet season = 0)

^a^ Households with a greater number of people younger than 18 years old than older than 18 = 1; households with a greater number of people 18 years old and older than 18 years old = 0.

## RESULTS

### Prevalence of wildlife use

**FIGURE 3:**
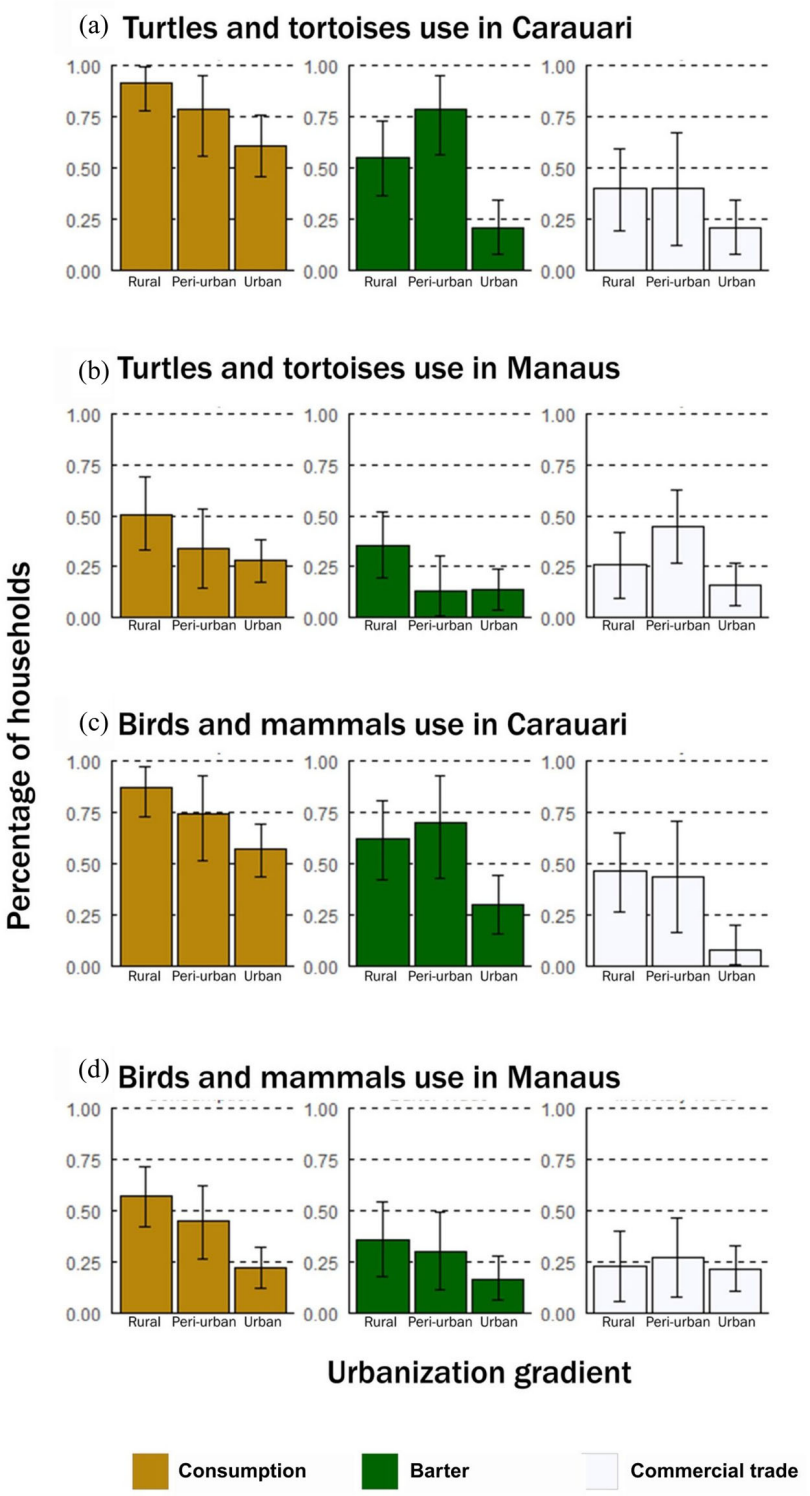
Percentage of households that used turtle and tortoise meat in (a) Carauari, a small town, and (b) Manaus, a large city, and that used birds and mammal meat in (c) Carauari and (d) Manaus across the gradient of urbanization during the dry season (June–December) of 2021 (error bars, 95% credible intervals).

For clarity, we present results for the dry season, when wildlife use was higher (Figure 3) (see Appendix S6 for the wet season). Wildlife consumption, barter, and commercial trade occurred across the urbanization categories (Figure 3). Wildlife use was higher for rural than urban households. Percentage of urban households that consumed (Manaus 22%, Carauari 57%), bartered (17% in Manaus, 30% in Carauari), and traded (Manaus 21%, Carauari 7%) meat from mammals and birds and that consumed (Manaus 28%, Carauari 61%), bartered (Manaus 14%, Carauari 20%), and traded (Manaus 16%, Carauari 21%) chelonians was not negligible. The percentage of peri‐urban households using wildlife did not differ from rural households. However, chelonians were bartered (79%, 95% confidence interval [CI] 57–95) and traded (44%, 95% CI 26–62) by a greater percentage of peri‐urban than urban households (barter in urban Carauari 20%, 95% CI 8–34; trade in urban Manaus 16%, 95% CI 6–26). In Carauari, a greater percentage of rural households traded mammal and bird meat (46%, 95% CI 26–65) than urban households (7%, 95% CI 0.4–19). The percentage of households that consumed wildlife was higher in Carauari than in Manaus (Figure 3). The percentage of peri‐urban households that bartered chelonians was greater in Carauari (79%, 95% CI 57–95) than in Manaus (13%, 95% CI 1–30). The percentage of households that traded wild meat did not differ among the municipalities sampled.

**FIGURE 4:**
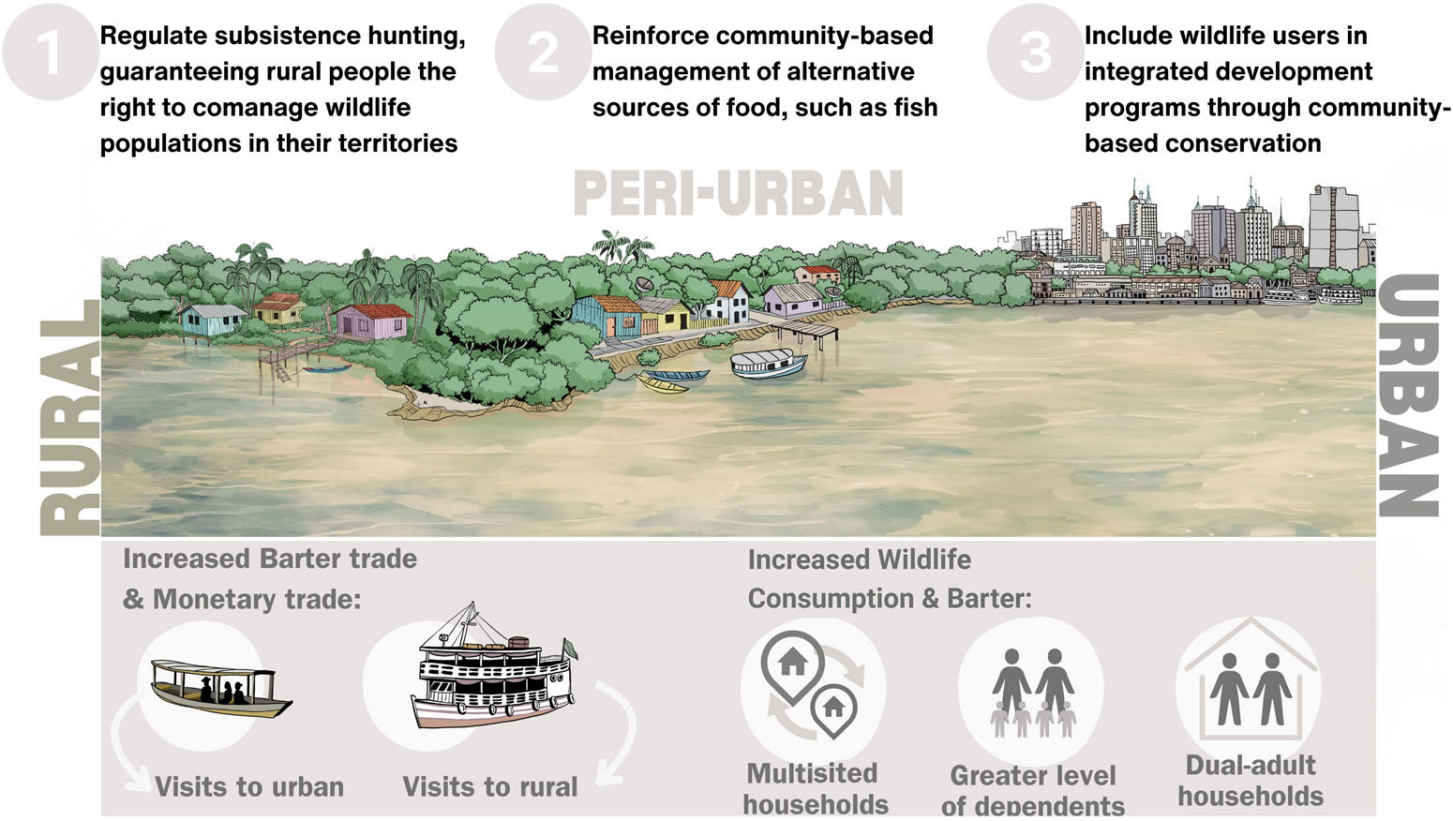
Effects of social mechanisms on wildlife use across the rural‐to‐urban gradient in the Brazilian Amazon and associated policy implications for participatory wildlife conservation. At the bottom are key social mechanisms affecting wildlife use: increased barter and commercial wildlife trade associated with visits between rural and urban areas; increased wildlife consumption and barter associated with multisited households (households accessing both urban and rural resources); higher dependency levels (ratio of minors to working adults); and dual‐adult households. Based on these mechanisms, 3 policy implications are proposed (top): regulate subsistence hunting, strengthen community‐based management of alternative food sources, and integrate wildlife users into development programs. Illustration by Norberto T. Ferreira.

The percentage of households that consumed mammals and birds was higher than the percentage of households that traded mammals and bird meat in rural Manaus and rural and urban Carauari (Figure 3). The percentage of households that consumed chelonians was higher than the percentage of households that traded chelonians in rural and urban Carauari (Figure 3). Peri‐urban wildlife use did not vary across uses (Figure 3). Finally, the percentage of households using wildlife did not vary by season (Figure 3, Appendix S6).

### Urbanization effects on social factors

Access to urban markets and rural resources was affected by the rural–urban gradient. Number of urban visits by households from rural and peri‐urban areas of Manaus was 30% greater than in Carauari (incidence rate ratio [IRR] 1.30, 95% CI 1.03–1.64), while the number of rural visits by urban households of Manaus was 75.8% lower than visits by urban households of Carauari (IRR 0.24, 95% CI 0.15–0.39). Finally, the number of urban visits by peri‐urban households was 3 times greater than rural households (IRR 3.02, 95% CI 2.42–3.77).

Nineteen percent of households were multisited; 63.3% had a primary urban residence. The number of visits to rural areas by multisited households with urban primary residence was 4‐fold greater than by single‐sited households (IRR 4.78, 95% CI 2.79–8.18). Number of visits to urban areas by rural households was not associated with residence status (Appendix S15).

The probability of being a dual‐adult household was 59% lower in Manaus (odds ratio [OR] 0.41, 95% CI 0.27–0.61) than in the small town. The odds of being a dual‐adult household were 60% greater for rural (OR 1.60, 95% CI 1.06–2.43) and more than 3 times greater for peri‐urban (OR 3.25, 95% CI 1.78–5.94) than urban households.

The prevalence of dependents over working‐age adults was strongly associated with municipality and urbanization categories. The odds of having a greater number of dependents over working‐age adults in the household was 48% lower in Manaus (OR 0.54, 95% CI 0.39–0.75) than in Carauari. The odds of having a greater number of dependents over working‐age adults were higher for rural and peri‐urban households (OR 1.94, 95% CI 1.34–2.82 rural and 1.26–3.4 peri‐urban) than urban households.

### Wild meat consumption

The urbanization categories and residence status were associated with wild meat consumption. Rural households consumed meat from mammals and birds 7 times more (OR 7.56, 95% CI 3.94–15.02) and chelonians 13 times more (OR 13.04, 95% CI 5.87–30.26) than urban households. Peri‐urban households consumed mammals and bird meat 3 times more (OR 3.42, 95% CI 1.64–7.23) and chelonians 6 times more (OR 6.64, 95% CI 2.82–16.73) than urban households. Multisited households consumed 2 times more meat from mammals and birds than single‐sited households (OR 2.51, 95% CI 1.38–4.54). Residence status did not affect chelonian consumption (Appendix S15). Living in Manaus was associated with 86% lower amount of meat from mammals and birds consumed (OR 0.14, 95% CI 0.07–0.25) and with 96% lower number of chelonians consumed (OR 0.04, 95% CI 0.02–0.1) than living in Carauari (Appendix S15). The amount of mammal and bird meat consumed was 55% greater (OR 1.55, 95% CI 1.05–2.34) and of chelonians was 7‐fold greater in dry (OR 7.09, 95% CI 3.91–14.04) than in the wet season.

As the number of rural visits by urban households increased by 1 SD (equivalent to 6.56 visits/season), the quantity of mammals and birds consumed increased by 36% (OR 1.36, 95% CI 1.08–1.77) and of chelonians increased almost 3‐fold (OR 2.94, 95% CI 1.69–4.83). Market access was not associated with wild meat consumption (Appendix S15). Level of dependency was positively associated with chelonians consumption. For each increase of 1 SD (equivalent to 0.2 level of dependency), the quantity of chelonians consumed increased by 42% (OR 1.42, 95% CI 1.08–1.97). Chelonian consumption was higher during dry than wet season (Appendix S15). The remaining variables included in the model were not associated with chelonian consumption (Appendix S15).

### Wild meat barter

The urbanization categories were associated with wild meat barter, residence status affected barter of mammals and birds, and season affected chelonian barter. Rural households bartered 82‐fold more mammal and bird meat (OR 82.72, 95% CI 19.36–554.18) and 5‐fold more chelonians (OR 5.12, 95% CI 2.02–12.95) than urban households. Peri‐urban households bartered 43 times more meat from mammals and birds (OR 43.81, 95% CI 9.46–298.95) and over 5 times more chelonians (OR 5.77, 95% CI 2.26–15.96) than urban households. Multisited households bartered 86% less mammal and bird meat than single‐sited households (OR 0.14, 95% CI 0.03–0.47). Households in Manaus bartered 99% less mammal and bird meat (OR 0.01, 95% CI 0.001–0.07) and 77% fewer chelonians (OR 0.23, 95% CI 0.08–0.54) than households in Carauari. More chelonians were bartered in the dry season (Appendix S15).

Among the factors assessed, dual‐adult households and market access were positively associated with the quantity of mammal and bird meat bartered. Dual‐adult households bartered 10 times more mammal and bird meat than single‐adult households (OR 0.13, 95% CI 1.42–167.63). An increase of 1 SD in the number of visits to the urban area (equivalent to 21.76 visits per season) was associated with 4‐fold more mammal and bird meat bartered (OR 4.12, 95% CI 1.11–17.91). As the number of dependents increased by 1 SD, the number of chelonians bartered increased by 53% (OR 1.53, 95% CI 1.12–2.05). Finally, the quantity of chelonians bartered in the dry season was greater than in the wet season (Appendix S15).

### Wild meat commercial trade

Urbanization effects on trade depended on the type of wildlife. The quantity of mammals and birds traded was 8 times greater for rural households than urban households (OR 8.35, 95% CI 2.62–34.59). The number of chelonians traded was 70% lower for households in Manaus than in Carauari (OR 0.30, 95% CI 0.11–0.72).

The level of dependency affected the quantity of mammals and birds commercially traded, whereas market access affected chelonian trade. The amount of mammal and bird meat traded was 52% greater for every increase of 0.2 in dependency level (OR 1.52, 95% CI 1.06–2.12). The quantity of chelonians traded was 2 times greater for every increase in 21.76 visits/season to urban areas (OR 2.39, 95% CI 1.04–6.55). The dry season was associated with higher commercial trade than the wet season, and this relationship was stronger for chelonians than mammals and birds (Appendix S15).

## DISCUSSION

We investigated how urbanization was associated with wildlife use. Our findings have implications for the inclusion of urban, peri‐urban, and rural populations in people‐centered conservation in the Brazilian Amazon. Wildlife use was widespread across the urbanization gradient, and wild meat use was associated with urbanization, municipality, residence status, and social factors. Although urbanization in Carauari (a small town) does not mirror urbanization in Manaus (a big city) exactly, the comparison provides insights into how social factors may influence wildlife use based on varying degrees of urbanization across these sites. The quantity of wild meat used was greater for rural and peri‐urban households than for urban households. However, urban demand for wild meat was substantial, highlighting the persistence of rural practices in urban contexts, especially in small towns. In Carauari, wildlife use was predominantly influenced by rural areas, whereas in Manaus, the urban area shaped rural and peri‐urban access to markets. This led to an increase in barter of species of lower market value (i.e., mammals and birds) and in commercial trade of species of higher market value (i.e., chelonians). Urban wild meat use increased as rural access by urban or multisited households increased. Finally, peri‐urban household proximity to wildlife and urban markets resulted in greater wildlife trade levels, especially closer to Manaus. These interlinked spatial and social processes have important, but largely overlooked, implications for wildlife policies.

### Wildlife trade and the urban frontier

We uncovered evidence of wildlife trade occurring in households across large and small urban areas. An average of 21% (7–34% Carauari) and 16% (6–26% Manaus) of urban households participated in trade. Urban households are engaged in transactions with other households, adding complexity to monitoring wild meat trade and enforcing regulations (Hughes et al., 2023). Participation of peri‐urban households in wildlife trade was substantial. Peri‐urban households engaged in diverse economic activities, often relying on access to both rural and urban markets. Their dynamic livelihood strategies and geographic position facilitate trade, and their involvement in the urban economy boosts wild meat demand (Torreset al., 2022). Peri‐urban households visited urban areas more often than they visited rural areas, suggesting stronger urban ties. Any conservation strategy concerning wildlife use should consider dynamics occurring in peri‐urban areas, including how inhabitants access wild meat.

### Embedded urbanization and urban‐to‐rural connections

We hypothesized that municipality size and household residence status are associated with access to wild meat and wildlife use, after accounting for urbanization categories. We found that small‐town households had higher rural access and higher wildlife use than Manaus residents, which is consistent with wildlife use literature in Amazonia (Torreset al., 2022; Chaves et al., 2017; Parry et al., 2010). In Manaus, however, urban access was greater than in Carauari. The diverse associations between rural access in small towns and market access in large cities stem from historical connectivity patterns among Amazonian cities and the process of “embedded urbanization” (Hecht et al., 2021). Small towns exhibit a subregional hierarchy, characterized by strong connections to local markets and natural resource exploitation (Guedes et al., 2009), suggesting a greater connection between small‐town inhabitants and wild meat. In contrast, large cities likely drive access across other urban dimensions, attracting people to markets and labor (Kanai, 2014).

Residence status had distinct effects on wildlife use. Multisited residents were consumers rather than intermediaries in wildlife barter, implying a straightforward network for wildlife products along the rural–urban continuum. This contrasts with the intricate and branched structure observed in other wildlife trade networks (e.g., commercial trade [Pantoja‐Lima et al., 2014]). Moreover, multisited residence increased rural access by urban households without a corresponding increase in urban access by rural households. These findings imply people's mobility across the urban–rural frontier by their residence status shapes access to wildlife, as evidenced elsewhere (Torreset al., 2022).

### Social factors affecting access to and use of wildlife

Visits to rural and urban areas, which are secondary effects of urbanization (Christiaensen & Todo, 2014), distinctly shaped wildlife use and could lead to an increase in wild meat consumption in urban areas and drive barter and commercial trade in rural and peri‐urban regions. Chelonian trading increased as market access increased. Mammals and birds, however, were bartered, a finding that contributes to evidence of a specific role for certain species. Although types of wildlife use for particular species have been identified in urban settings (Chaves et al., 2023), residents in rural and peri‐urban areas likely favor bartering cheaper food items, reserving commercial transactions for economically valuable species, such as chelonians (El Bizri et al., 2020). Thus, household wildlife use involves social and economic mechanisms, and the species affects the type of use.

Intrahousehold factors also influenced wildlife use. Dual‐adult households consumed and bartered more bird and mammal meat than single‐adult households. This suggests that dual‐adult households tend to access wildlife and facilitate others’ access to wildlife through food‐sharing practices. With 2 adults, the capacity to engage in hunting or wildlife acquisition activities, either directly or through social networks, is enhanced (Wood & Marlowe, 2013). Conversely, it suggests that single‐adult households are vulnerable to food shortages if they rely on wild meat or that single‐sited households may rely on domesticated meat. Single‐adult households’ participation in urban markets was higher than dual‐adult households, possibly because of their prevalence in Manaus. This may be associated with reduced access to wild meat.

High rates of barter among households with greater dependency levels might mean greater reliance on social networks (Barbieri et al., 2021). In fact, participating in wild meat barter likely extends benefits beyond intrahousehold food security to community well‐being and expansion of rural–urban networks (Carigiano Torres et al., 2022; Chaves et al., 2023). In addition, wild meat sharing can be a safety net during periods of food scarcity (Valle Nunes et al., 2019). Then, households with more dependents might cope with food availability fluctuations by bartering meat, consequently fostering reciprocity outside the household.

We also found that household dependency level increased chelonian consumption and barter. This finding challenges the association of chelonian consumption with wealth, despite expectations of economic strain in such households. This relationship warrants further investigation, particularly because we did not detect an effect of dependency level on trade. It is possible, however, that people are investing efforts to maintain chelonian barter, thereby increasing the availability of these species in the household (El Bizri et al., 2023).

### Integrating the urbanization gradient in participatory wildlife policies

Conservation in Brazil highlights the participation of people in research, yet it often lacks frameworks to effectively integrate wildlife users in decision‐making (Bragagnolo et al., 2019). Aside from policies supporting Indigenous wildlife use, fair and effective interventions are rare (Antunes et al., 2019). For example, although 84% of protected areas in the Brazilian Amazon are comanaged by traditional communities, a review of 30 management plans showed that less than half considered people legitimate users (Lemos et al., 2023). Additionally, efforts to include people in wildlife policy are often biased toward rural areas (i.e., extractive reserves [ICMBIO, 2002]) and toward specific taxa (i.e., caimans [CEMAAM, 2011] and chelonians [ICMBIO, 2022]). Plans to address wildlife stewardship must be accompanied by a transformation in environmental governance with a framework that addresses the relationships between rural and urban populations, prioritizes harvesting of resilient species, and helps alleviate poverty (Parry et al., 2014). Therefore, a structural shift is required toward a sustainable and just wildlife use sector in conservation. We propose 3 recommendations to promote this shift that are based on wildlife use as ongoing across the urbanization gradient, despite legal restrictions.

We applied a social justice framework that places human rights at the center of conservation (Milner‐Gulland, 2024). Hunting regulations in the Brazilian Amazon must accommodate the fundamental role of hunting in rural subsistence economies (Antunes et al., 2019). The prevalence of rural households consuming wild meat, ranging from 50% (in Manaus) to 92% (in Carauari) in our study, underscores the critical subsistence role of wild meat. In rural Amazonia, wild meat consumption alleviates child malnutrition, enhances food security, and is a vital resource for populations facing economic and social constraints (Bachmann et al., 2019; Carigiano Torres et al., 2022; Nunes et al., 2019). Despite its importance to 26% of the Amazonian population in Brazil (IBGE, 2010), wild meat use remains largely unregulated, leaving people subject to contradictory rules and policies (Van Vliet, 2018). Considering the prevalence of rural wild meat consumption, we recommend the adoption of an inclusive legal framework that regulates subsistence hunting and guarantees rural people the right to comanage wildlife populations in their territories (Figure 4, number 1).

Ensuring sustainable rural wild meat use requires complementary strategies that encourage livelihood diversification and uphold territorial sovereignty (Wicander & Coad, 2018). Therefore, we recommend that conservation policies encompass Amazonian food systems comprehensively and prioritize reinforcement of community‐based management approaches for alternative wild food sources, such as fish (Figure 4, number 2). Fish are an abundant and reliable food and nutrient source for Amazonian communities compared with wild meat (Tregidgo et al., 2020). Thus, coupled wildlife–fish stock management should support rural communities, particularly when populations of consumed species fluctuate. Moreover, fish management initiatives will likely benefit terrestrial species using these managed systems due to the territorial protection they promote (Campos‐Silva et al., 2018). Integrating fish and wildlife management in a community‐based framework would significantly enhance cooperation and collective action, thereby fostering robust local institutions capable of deliberating on wildlife use and rural sustainable development.

Peri‐urban areas emerged as significant contributors to the integration of rural wildlife stocks in urban markets, facilitating wildlife barter and commercial trade, particularly in Manaus where demand for wildlife is likely unsustainable (Chaves et al., 2021). Peri‐urban regions are focal points for market integration and infrastructural development as urban areas expand (Hutchings et al., 2022). However, peri‐urban expansion often occurs without adequate planning (Allen, 2003). Given that the growth of peri‐urban areas can accelerate demand for wild meat and contribute to the depletion of wildlife populations near urban centers (Sampaio et al., 2022) and that urban hunters may also harvest wildlife for other purposes not addressed here (i.e., sport; Oliveira et al., 2023), we recommend including wildlife users in integrated development programs through community‐based conservation efforts to mitigate rural and peri‐urban participation in wildlife markets (Figure 4, number 3). Consequently, integrated wildlife conservation plans should encompass broader considerations related to urban expansion and local development policies. Municipal master plans, for instance, could significantly contribute to wildlife conservation efforts by adopting a regional approach that includes people across the urbanization spectrum, which could also prevent unsustainable urban growth. We recognize that a more nuanced, multidimensional approach (e.g., incorporating socioeconomic, infrastructural, and ecological variables) would provide richer insights into the urban–rural continuum effects on wildlife use.

Bridging the historical divide between urban and rural conservation strategies is paramount. Aside from a 2006 attempt to cocreate policy for wildlife use, known as the “Amazonian Wildlife Policy” (IBAMA, 2006), few conservation initiatives proposed structural changes to enhance sustainable rural–urban intersections. Notable exceptions include regulations on sport hunting (Law 10,056;94) and on hunting invasive species (IBAMA Normative Instruction 03/2013; El Bizri et al., 2015). Despite these localized efforts, rural and urban wildlife users should play roles in decision‐making processes so that policies that reduce dependence on wildlife and address food sovereignty and well‐being are developed. Rural institutions have shown adaptability in addressing the effects of urbanization, such as market integration (Hecht et al., 2021). For urban residents, wildlife policies should focus on voluntary demand reduction initiatives and target enforcement of unsustainable trade markets. We found that populations in the peri‐urban areas of Manaus sustain illegal trade, which reinforces people's vulnerability. Critically, integrated yet targeted policies should produce clear conservation outcomes that encompass social and ecological dimensions and focus on the inclusion of vulnerable, yet overlooked, urban, peri‐urban, and rural residents. Ultimately, a collaborative approach recognizing the interconnectedness of rural and urban spheres can create sustainable pathways for an urbanizing Amazonia.

## Supporting information



Supporting Information

## References

[cobi70049-bib-0001a] Allen, A. (2003). Environmental planning and management of the peri‐urban interface: perspectives on an emerging field. Environment & Urbanization, 15(1), 13.

[cobi70049-bib-0001] Antunes, A. P. , Rebêlo, G. H. , Pezzuti, J. C. B. , Vieira, M. A. R. D. M. , Constantino, P. D. A. L. , Campos‐Silva, J. V. , Fonseca, R. , Durigan, C. C. , Ramos, R. M. , Amaral, J. V. D. , Camps Pimenta, N. , Ranzi, T. J. D. , Lima, N. A. S. , & Shepard, G. H. (2019). A conspiracy of silence: Subsistence hunting rights in the Brazilian Amazon. Land Use Policy, 84, 1–11.

[cobi70049-bib-0002] Bachmann, M. E. , Junker, J. , Mundry, R. , Nielsen, M. R. , Haase, D. , Cohen, H. , Kouassi, J. A. K. , & Kühl, H. S. (2019). Disentangling economic, cultural, and nutritional motives to identify entry points for regulating a wildlife commodity chain. Biological Conservation, 238, Article 108177.

[cobi70049-bib-0003] Barbieri, A. F. , Guedes, G. R. , & Onofre Dos Santos, R. (2021). Land use systems and livelihoods in demographically heterogeneous frontier stages in the amazon. Environmental Development, 38, Article 100587.

[cobi70049-bib-0004] Becker, B. (2005). Geopolítica da Amazônia. Estududos Avançados, 19(53), 71–86.

[cobi70049-bib-0005] Blair, G. , Imai, K. , & Zhou, Y.‐Y. (2015). Design and analysis of the randomized response technique. Journal of the American Statistical Association, 110, 1304–1319.

[cobi70049-bib-0006] Bragagnolo, C. , Gama, G. M. , Vieira, F. A. S. , Campos‐Silva, J. V. , Bernard, E. , Malhado, A. C. M. , Correia, R. A. , Jepson, P. , De Carvalho, S. H. C. , Efe, M. A. , & Ladle, R. J. (2019). Hunting in Brazil: What are the options? Perspectives in Ecology and Conservation, 17, 71–79.

[cobi70049-bib-0007] Brasil . (1967). Dispõe sobre a proteção à fauna e dá outras providências . http://www.planalto.gov.br/ccivil_03/leis/L5197.htm

[cobi70049-bib-0008] Brasil . (1998). Dispõe sobre as sanções penais e administrativas derivadas de condutas e atividades lesivas ao meio ambiente, e dá outras providências . http://www.planalto.gov.br/ccivil_03/leis/L5197.htm

[cobi70049-bib-0009] Brooks, M. E. , Kristensen, K. , Benthem, K. J. V. , Magnusson, A. , Berg, C. W. , Nielsen, A. , Skaug, H. J. , Mächler, M. , & Bolker, B. M. (2017). glmmTMB balances speed and flexibility among packages for zero‐inflated generalized linear mixed modeling. The R Journal, 9, 378–400.

[cobi70049-bib-0010] Browder, J. O. , & Godfrey, B. J. (1997). Rainforest cities: Urbanization, development, and globalization of the Brazilian Amazon. Columbia University Press.

[cobi70049-bib-0011] Campos‐Silva, J. V. , Hawes, J. E. , Andrade, P. C. M. , & Peres, C. A. (2018). Unintended multispecies co‐benefits of an Amazonian community‐based conservation programme. Nature Sustainability, 1, 650–656.

[cobi70049-bib-0012] Carignano Torres, P. , Morsello, C. , Orellana, J. D. Y. , Almeida, O. , De Moraes, A. , Chacón‐Montalván, E. A. , Pinto, M. A. T. , Fink, M. G. S. , Freire, M. P. , & Parry, L. (2022). Wildmeat consumption and child health in Amazonia. Scientific Reports, 12, Article 5213. 35388037 10.1038/s41598-022-09260-3 PMC8986765

[cobi70049-bib-0013] Carignano Torres, P. , Morsello, C. , & Parry, L. (2022). Rural–urban mobility influences wildmeat access and consumption in the Brazilian Amazon. Oryx, 56, 864–876.

[cobi70049-bib-0014] CEMAAM . (2011). Estabelece os critérios técnicos para o manejo de jacaré, oriundo de unidades de conservação de uso sustentável do Estado do Amazonas . Author.

[cobi70049-bib-0015] Chaves, W. , Carignano Torres, P. , & Parry, L. (2023). The species‐specific role of wildlife in the Amazonian food system. Ecology and Society, 28, Article 28.

[cobi70049-bib-0016] Chaves, W. A. , Valle, D. , Tavares, A. S. , Morcatty, T. Q. , & Wilcove, D. S. (2021). Impacts of rural to urban migration, urbanization, and generational change on consumption of wild animals in the Amazon. Conservation Biology, 35, 1186–1197. 33124717 10.1111/cobi.13663

[cobi70049-bib-0017] Chaves, W. A. , Valle, D. , Tavares, A. S. , von Mühlen, E. M. , & Wilcove, D. S. (2021). Investigating illegal activities that affect biodiversity: The case of wildlife consumption in the Brazilian Amazon. Ecological Applications, 31, Article e02402. 34233059 10.1002/eap.2402

[cobi70049-bib-0018] Chaves, W. A. , Wilkie, D. S. , Monroe, M. C. , & Sieving, K. E. (2017). Market access and wild meat consumption in the central Amazon, Brazil. Biological Conservation, 212, 240–248.

[cobi70049-bib-0019] Christiaensen, L. , & Todo, Y. (2014). Poverty reduction during the rural–urban transformation—The role of the missing middle. World Development, 63, 43–58.

[cobi70049-bib-0020] El Bizri, H. R. , Morcatty, T. Q. , Ferreira, J. C. , Mayor, P. , Vasconcelos Neto, C. F. A. , Valsecchi, J. , Nijman, V. , & Fa, J. E. (2023). Social and biological correlates of wild meat consumption and trade by rural communities in the Jutaí River Basin, Central Amazonia. Journal of Ethnobiology, 40, 183–201.

[cobi70049-bib-0021] El Bizri, H. R. , Morcatty, T. Q. , Lima, J. J. S. , & Valsecchi, J. (2015). The thrill of the chase: Uncovering illegal sport hunting in Brazil through YouTube™ posts. Ecology and Society, 20, Article 30.

[cobi70049-bib-0022] El Bizri, H. R. , Morcatty, T. Q. , Valsecchi, J. , Mayor, P. , Ribeiro, J. E. S. , Vasconcelos Neto, C. F. A. , Oliveira, J. S. , Furtado, K. M. , Ferreira, U. C. , Miranda, C. F. S. , Silva, C. H. , Lopes, V. L. , Lopes, G. P. , Florindo, C. C. F. , Chagas, R. C. , Nijman, V. , & Fa, J. E. (2020). Urban wild meat consumption and trade in central Amazonia. Conservation Biology, 34, 438–448. 31538670 10.1111/cobi.13420

[cobi70049-bib-0023] Elmqvist, T. , Andersson, E. , Mcphearson, T. , Bai, X. , Bettencourt, L. , Brondizio, E. , Colding, J. , Daily, G. , Folke, C. , Grimm, N. , Haase, D. , Ospina, D. , Parnell, S. , Polasky, S. , Seto, K. C. , & Van Der Leeuw, S. (2021). Urbanization in and for the Anthropocene. Urban Sustainability, 1, Article 6.

[cobi70049-bib-0024] Gonçalves, J. , Gomes, M. C. , Ezequiel, S. , Moreira, F. , & Loupa‐Ramos, I. (2017). Differentiating peri‐urban areas: A transdisciplinary approach towards a typology. Land Use Policy, 63, 331–341.

[cobi70049-bib-0025] Guedes, G. , Costa, S. , & Brondízio, E. (2009). Revisiting the hierarchy of urban areas in the Brazilian Amazon: A multilevel approach. Population and Environment, 30, 159–192. 23129877 10.1007/s11111-009-0083-3 PMC3488306

[cobi70049-bib-0026] Hecht, S. , Schmink, M. , Abers, R. , Assad, E. D. , Bebbington, D. H. , Brondizio, E. S. , Costa, F. A. , Calisto, A. M. D. , Fearnside, P. M. , Garrett, R. , Heilpern, S. , McGrath, D. , Oliveira, G. , Pereira, H. S. , & Pinedo‐Vazquez, M. (2021). Chapter 14: Amazon in Motion: Changing politics, development strategies, peoples, landscapes, and livelihoods. In C. Nobre , A. Encalada , E. Anderson , F. H. Roca Alcazar , M. Bustamante , C. Mena , M. Peña‐Claros , G. Poveda , J. P. Rodriguez , S. Saleska , S. Trumbore , A. L. Val , L. Villa Nova , R. Abramovay , A. Alencar , C. Rodríguez Alzza , D. Armenteras , P. Artaxo , S. Athayde , … G. Zapata‐Ríos (Eds.), Amazon Assessment Report 2021 (1st ed.). UN Sustainable Development Solutions Network (SDSN). https://www.theamazonwewant.org/amazon‐assessment‐report‐2021/

[cobi70049-bib-0027] Hutchings, P. , Willcock, S. , Lynch, K. , Bundhoo, D. , Brewer, T. , Cooper, S. , Keech, D. , Mekala, S. , Mishra, P. P. , Parker, A. , Shackleton, C. M. , Venkatesh, K. , Vicario, D. R. , & Welivita, I. (2022). Understanding rural–urban transitions in the Global South through peri‐urban turbulence. Nature Sustainability, 5, 924–930.

[cobi70049-bib-0027a] Hughes, L. J. , Morton, O. , Scheffers, B. R. , & Edwards, D. P. (2023). The ecological drivers and consequences of wildlife trade. Biological reviews of the Cambridge Philosophical Society, 98(3), 775–791. 10.1111/brv.12929 36572536

[cobi70049-bib-0028] IBGE . (2010). Cidades@ . http://www.cidades.ibge.gov.br/xtras/home.php

[cobi70049-bib-0029] IBGE . (2022). Population . https://www.ibge.gov.br/en/statistics/social/population.html

[cobi70049-bib-0030] ICMBio . (2002). Estabelece normas para o uso sustentável da fauna silvestre brasileira autóctone não ameaçada de extinção, tradicionalmente utilizada pelas populações tradicionais em Reservas Extrativistas . Author.

[cobi70049-bib-0031] ICMBio . (2022). Estabelece normas e procedimentos para o manejo comunitário de quelônios das espécies tartaruga‐da‐amazônia (Podocnemis expansa) e tracajá (Podocnemis unifilis), em Floresta Nacional (Flona), Reserva Extrativista (Resex) e Reserva de Desenvolvimento Sustentável (RDS) federais, nas áreas de ocorrência natural das espécies, e dá outras providências . Author.

[cobi70049-bib-0032] Ingram, D. J. , Coad, L. , Milner‐Gulland, E. J. , Parry, L. , Wilkie, D. , Bakarr, M. I. , Benítez‐López, A. , Bennett, E. L. , Bodmer, R. , Cowlishaw, G. , El Bizri, H. R. , Eves, H. E. , Fa, J. E. , Golden, C. D. , Iponga, D. M. , Minh, N. V. , Morcatty, T. Q. , Mwinyihali, R. , Nasi, R. , … Abernethy, K. (2021). Wild meat is still on the menu: Progress in wild meat research, policy, and practice from 2002 to 2020. Annual Review of Environment and Resources, 46, 221–254.

[cobi70049-bib-0033] Instituto Brasileiro do Meio Ambiente e dos Recursos Naturais Renováveis (IBAMA) . (2006). Politica de fauna silvestre da Amazônia . Author.

[cobi70049-bib-0034] Intergovernmental Science‐Policy Platform on Biodiversity and Ecosystem Services (IPBES) . (2019). Summary for policymakers of the global assessment report on biodiversity and ecosystem services of the Intergovernmental Science‐Policy Platform on Biodiversity and Ecosystem Services. IPBES Secretariat. 10.5281/zenodo.3553579

[cobi70049-bib-0035] Kanai, J. M. (2014). On the peripheries of planetary urbanization: Globalizing Manaus and its expanding impact. Environment and Planning D: Society and Space, 32, 1071–1087.

[cobi70049-bib-0036] Lemos, L. P. , Ferreira, D. S. , Oliveira, M. A. , Morcatty, T. Q. , Antunes, A. P. , de Souza Jesus, A. , El Bizri, H. R. , Pezzuti, J. , Ramos, R. M. , Santos‐Fita, D. , & Pimenta, N. C. (2023). Subsistence hunting and wild meat trade in Brazilian Amazonia. In W. R. Spironello , A. A. Barnett , J. W. Lynch , P. E. D. Bobrowiec , & S. A. Boyle (Eds.), Amazonian mammals (pp. 241–274). Springer International Publishing. https://link.springer.com/10.1007/978‐3‐031‐43071‐8_9

[cobi70049-bib-0037] Londres, M. , Salk, C. , Andersson, K. P. , Tengö, M. , Brondizio, E. S. , Russo Lopes, G. , Siani, S. M. O. , Molina‐Garzón, A. , Sonetti‐González, T. , Montoya, D. R. , Futemma, C. , De Castro, F. , & Tourne, D. C. M. (2023). Place‐based solutions for global social‐ecological dilemmas: An analysis of locally grounded, diversified, and cross‐scalar initiatives in the Amazon. Global Environmental Change, 82, Article 102718.

[cobi70049-bib-0038] Milner‐Gulland, E. J. (2024). Now is the time for conservationists to stand up for social justice. PLoS Biology, 22, Article e3002657. 38857193 10.1371/journal.pbio.3002657 PMC11164339

[cobi70049-bib-0039] Nielsen, M. R. , Meilby, H. , Smith‐Hall, C. , Pouliot, M. , & Treue, T. (2018). The importance of wild meat in the Global South. Ecological Economics, 146, 696–705.

[cobi70049-bib-0040] Nunes, A. V. , Peres, C. A. , Constantino, P. , Santos, B. A. , & Fischer, E. (2019). Irreplaceable socioeconomic value of wild meat extraction to local food security in rural Amazonia. Biological Conservation, 236, 171–179.

[cobi70049-bib-0041] Oliveira, M. A. , El Bizri, H. R. , Morcatty, T. Q. , Braga‐Pereira, F. , Fa, J. E. , Messias, M. R. , & Da Costa Doria, C. R. (2023). The role of religion, wealth, and livelihoods in the hunting practices of urban and rural inhabitants in Western Amazonia. Human Ecology, 51, 1239–1252. 10.1007/s10745-023-00467-0

[cobi70049-bib-0042] Padoch, C. , Brondizio, E. , Costa, S. , Pinedo‐Vasquez, M. , Sears, R. R. , & Siqueira, A. (2008). Urban forest and rural cities: Multi‐sited households, consumption patterns, and forest resources in Amazonia. Ecology and Society, 13, Article 2.

[cobi70049-bib-0043] Pantoja‐Lima, J. , Aride, P. H. , De Oliveira, A. T. , Félix‐Silva, D. , Pezzuti, J. C. , & Rebêlo, G. H. (2014). Chain of commercialization of *Podocnemis* spp. turtles (Testudines: Podocnemididae) in the Purus River, Amazon basin, Brazil: Current status and perspectives. Journal of Ethnobiology and Ethnomedicine, 10, Article 8. 24467796 10.1186/1746-4269-10-8 PMC3933064

[cobi70049-bib-0044] Parry, L. , Barlow, J. , & Pereira, H. (2014). Wildlife harvest and consumption in Amazonia's urbanized wilderness. Conservation Letters, 7, 565–574.

[cobi70049-bib-0045] Parry, L. , Barlow, J. , & Peres, C. A. (2009). Allocation of hunting effort by Amazonian smallholders: Implications for conserving wildlife in mixed‐use landscapes. Biological Conservation, 142, 1777–1786.

[cobi70049-bib-0046] Parry, L. , Day, B. , Amaral, S. , & Peres, C. A. (2010). Drivers of rural exodus from Amazonian headwaters. Population and Environment, 32, 137–176.

[cobi70049-bib-0047] Phadke, A. (2020). Peri‐urbanization, Global South. In A. Kobayashi (Ed.), International encyclopedia of human geography (pp. 71–77). Elsevier. https://linkinghub.elsevier.com/retrieve/pii/B9780081022955106778

[cobi70049-bib-0048] Pinho, P. F. , Patenaude, G. , Ometto, J. P. , Meir, P. , Toledo, P. M. , Coelho, A. , & Young, C. E. F. (2014). Ecosystem protection and poverty alleviation in the tropics: Perspective from a historical evolution of policy‐making in the. Brazilian Amazon. Ecosystem Services, 8, 97–109.

[cobi70049-bib-0049] Plummer, M. (2003). JAGS: A program for analysis of Bayesian graphical models using Gibbs sampling. In K. Hornik , F. Leisch , & A. Zeileis (Eds.), Proceedings of the 3rd International Workshop on Distributed Statistical Computing (DSC 2003), Vienna, 20‐22 March 2003 (pp. 1–10). Technische Universität Wien.

[cobi70049-bib-0050] R Core Team . (2020). R: A language and environment for statistical computing . R Foundation for Statistical Computing. https://www.R‐project.org/

[cobi70049-bib-0051] Ribot, J. C. , & Peluso, N. L. (2003). A theory of access. Rural Sociology, 68, 153–181.

[cobi70049-bib-0052] Sampaio, R. , Morato, R. G. , Abrahams, M. I. , Peres, C. A. , & Chiarello, A. G. (2022). Physical geography trumps legal protection in driving the perceived sustainability of game hunting in Amazonian local communities. Journal for Nature Conservation, 67, Article 126175.

[cobi70049-bib-0053] Tregidgo, D. , Barlow, J. , Pompeu, P. S. , & Parry, L. (2020). Tough fishing and severe seasonal food insecurity in Amazonian flooded forests. People and Nature, 2, 468–482.

[cobi70049-bib-0054] Valle Nunes, A. , Guariento, R. D. , Santos, B. A. , & Fischer, E. (2019). Wild meat sharing among non‐indigenous people in the southwestern Amazon. Behavioral Ecology and Sociobiology, 73, Article 26.

[cobi70049-bib-0055] Van Vliet, N. (2018). “Bushmeat crisis” and “cultural imperialism” in wildlife management? Taking value orientations into account for a more sustainable and culturally acceptable wildmeat sector. Frontiers in Ecology and Evolution, 6, Article 112.

[cobi70049-bib-0056] Van Vliet, N. , Quiceno, M. P. , Cruz, D. , Jonhson Neves De Aquino, L. , Yagüe, B. , Schor, T. , Hernandez, S. , & Nasi, R. (2015). Bushmeat networks link the forest to urban areas in the trifrontier region between Brazil, Colombia, and Peru. Ecology and Society, 20, Article 21.

[cobi70049-bib-0057] Wicander, S. , & Coad, L. (2018). Can the provision of alternative livelihoods reduce the impact of wild meat hunting in West and Central Africa? Conservation and Society, 16, 441–458.

[cobi70049-bib-0058] Wickham, H. , François, R. , Henry, L. , & Müller, K. (2021). dplyr: A grammar of data manipulation . https://CRAN.R‐project.org/package=dplyr

[cobi70049-bib-0059] Wood, B. M. , & Marlowe, F. W. (2013). Household and kin provisioning by Hadza Men. Human Nature, 24, 280–317. 23813245 10.1007/s12110-013-9173-0

