# Supplementary material for: Including the urbanization gradient in people‐centered wildlife conservation in Amazonia
Source: Conserv Biol. 2025 Apr 28;39(3):e70049. doi: 10.1111/cobi.70049 (PMC12124168; doi:10.1111/cobi.70049)
Supplement: Supplementary file 1 — Supporting Information [file COBI-39-e70049-s001.docx]

**Appendix S1 – Detailed information on methods and results for quantity of wild meat used**

**Methods**

*Sampling design*

In urban Manaus, we randomly selected 50 points stratified by district, accounting for household density (Figure 2), and surveyed six randomly selected households closest to the point location. In urban Carauari, we randomly selected 159 points and sampled the closest household to the point location. For peri-urban and rural areas, we randomly selected riverine villages (i.e. the ones using the river and its streams to access the main urban center, including those that would occasionally use roads) and randomized the selection of sampled households according to village size. For villages smaller than 100 households, we counted the households and randomly selected at least eight for the surveys. For villages greater than 100 households, we randomly selected streets and surveyed one household in every four households in each street, until we reached eight surveys per village (Appendix S4).

*Household surveys*

*Applying the unrelated question design technique*: We used dominoes as randomizers, and they were placed in a non-transparent bag that was shaken by the participant prior to choosing one out of six domino pieces: four dominoes had two dots and would assign responses for the sensitive item, while two dominoes had one dot and would assign answers for the non-sensitive item. The participants would answer the survey questions based on which sensitive or non-sensitive item was randomly assigned to them. Only the participant could see the selected domino. Sensitive items in our study were meat from mammals and birds or from turtles and tortoises, while non-sensitive items were pasta, corn dishes, cassava, cooking oil, batteries, and toothbrushes. Since seasonality influences wildlife in the study region (Chaves et al. 2021), we asked survey questions about the dry season (ranging from June to November 2021 in Manaus, and from July to December 2021 in Carauari) and the wet season (ranging from December 2021 to May 2022 in Manaus, and from January to June 2022 in Carauari). Survey tools were tested in each municipality before being applied and are available at Appendix S2.

*Statistical models for wildlife use quantity*

Wildlife use quantity refers to the quantity consumed or traded from June 2021 to May 2022 period. For participants who reported using the items weekly, monthly, or throughout a given season, the reference number of days was 7, 30, and 180, respectively. We categorized use quantity into five categories (Appendix S5). We used histograms to explore the distribution of the data and then determine the optimal cut-off points that ensure equitable distribution of units across five quantity categories.

Let *yi* be an integer between 1 and 5 indicating the use quantity category of household *i*. We model *yi* using an ordered logistic regression model and we fit this model in a Bayesian framework for both the direct questions (used for the non-sensitive items) and indirect questions (used for wild meat use). Similar to what we did for the prevalence of wildlife use, we relied on 25,000 samples from the posterior distribution, after discarding the first 50,000 iterations as burn-in period.

Direct questions about non-sensitive items

We relied on an ordered logistic regression model in which we assume that the ordinal response variable *yi* for person *i* arises from the following distribution:

$${\gamma i}^{NSI}\sim Cat(\pi_{i}^{NSI})$$

Where $\pi_{i}^{NSI}$ is a vector of probabilities that sum to one for the non-sensitive item (NSI) (see Chaves et al. 2023 for more details).

Indirect questions about wildlife use

For the indirect question (IQ) on the sensitive item, $W_{i}^{IQ}$. If $W_{i}^{IQ}=1$ denoted the latent status of the household *i,* meaning that respondent *i* was answering about the sensitive item (SI). On the other hand, if $W_{i}^{IQ}=0$ the respondent was answering about the non-sensitive item (NSI). We assumed that:

$$W_{i}^{IQ}\sim Bernoulli (\frac{4}{6})$$

where 4/6 is the proportion of domino pieces with two dots, referring to the sensitive item whenever a participant drew a domino piece with two dots. We assumed that the ordinal response variable for the indirect question arises from:

$$Y_{i}^{IQ}\sim Cat(W_{i}^{IQ}\pi_{i}^{SI}+(1-W_{i}^{IQ})\pi^{NSI} )$$

where $\pi_{i}^{SI}$ is another vector of probabilities that sum to one, but for the sensitive item (SI) (see Chaves et al. 2023 for more details).

Estimating quantity of wild meat used by municipality, urbanization gradient, and season

We estimated the quantity used (number of kg or units used per month) by households in Manaus and Carauari using the models described above. Our covariates included municipality, urbanization gradient (rural, peri-urban, or urban), and season. These model specifications enabled the estimation of quantity used per household for each municipality, category of urbanization, and season.

*Factors associated with wildlife use*

We used ordered logistic regression models to look at additional factors associated with consumption, barter trade, and monetary trade of wild meat. Our first model included spatial factors: municipality (Manaus and Carauari), urbanization gradient, and residence status. The second model included social factors associated with wildlife use: single- or dual adult household and level of dependents. We opted to run separate models for rural access and market access because each included a different set of households. More specifically, a third model assessed how rural access, quantified as the number of visits to the rural area, influenced wildlife use. For this analysis, we only used data from urban households. A fourth model included only rural and peri-urban households and assessed how market access, quantified as the number of visits to the urban area, influenced wildlife use.

**Results**

*Quantity of wildlife used*

We present the percentage of households that used wildlife during the dry season, merging the fourth and fifth quantity category, for clarity (Figure 3). Results associated with other quantity categories and the wet season are presented in Appendix S7-S13). The quantity of chelonian meat consumed decreased across the rural-urban gradient, while the quantity of mammal and bird meat consumed was similar among rural, peri-urban, and urban zones (Figure 3). Barter trade of mammal and bird meat was performed by 90% (5.8%-99%) of peri-urban households in Carauari, but was similar among rural, and urban areas. The percentage of households that barter traded chelonians was minimal and similar among zones, seasons, and municipalities sampled. We also did not detect differences in the percentage of households that traded wild meat across the urbanization gradient, even though mammals and birds tend to be traded by a greater percentage of peri-urban households than in any other areas of both Manaus and Carauari. The percentage of households trading wildlife did not vary between seasons.

Quantity consumed of chelonians was almost threefold greater during the dry season than for the wet season (OR:2.95; CI:1.89-4.71) when accounting for social factors, and four times greater during the dry season than the wet season (OR = 4.19; 95% CI = 2.42 - 7.34) when accounting for the number of rural and peri-urban visits to the urban area.

**Appendix S2 – Survey instruments**

**INDIRECT Questionnaires - Access and use of wildlife**

| Participant Id:_____________ | ( ) Man ( ) Woman ( ) Other: _______ |  |
| --- | --- | --- |
| Municipality:______________ | District: __________________ | Ngbrd./Comnty:____________ |
| Date: ______/______/______ | Time (start) _______:_______ | Time (finish) _______:______ |
| Interviewer:_____________________________ Assistant: ______________________________ | | |

| **Part 1 - Indirect questions - EXAMPLE** |
| --- |

*I'd like to start by asking a few questions about the consumption, purchase, sale, and barter of food and other items. The questions include consumption, hunting, trading and selling wild animals and chelonians. As these are sensitive topics, people are sometimes worried about hunting, consuming, selling or trading these animals. So I'm going to use an indirect way of asking the questions. That way I won't get to know what you're talking about. So you can feel free to talk about it, but without giving me a direct answer.* ***The questions are not about what only you do, but about what everyone in your household, including you, does.*** *I'll give an example of how it works.*

*I have two types of food here. I will ask questions about your consumption of one of them. You'll answer me, but I won't know which one you're answering [SHOW BOTH ITEMS]*

| **Item 1: eat beans** |
| --- |
| **Item 2: eat chicken** |

We're going to use dominoes to decide which food you're going to answer about. Inside this bag there are 6 dominoes; two pieces have a value of 1 and four pieces have a value of 2 [ASK FOR PARTICIPANT TO CHECK THE PIECES]. You will shake the bag, put your hand inside the bag and, without looking, you will pick up a piece. If the value of the domino is 1, you answer the questions about item 1. If the value is 2, you answer the questions about item 2. Ok? Let's try an example.

Shake the bag and get a domino piece. Look at the result. The value [READ OUT THE VALUE] is out. The rule is, if the value is 1 (1 ball) you answer the questions about food 1 (beans). If it is 2 (2 balls), you answer the questions about food 2 (chicken). In that case, which food will you answer about? [READ ITEMS AGAIN]

[WAIT FOR THE PARTICIPANT TO ANSWER AND, DEPENDING ON THE ANSWER, CONFIRM OR CORRECT]

Now shake the bag again and get a domino piece. This time without showing me.

[REPEAT THE PROCESS OF TAKING A DOMINO PIECE, BUT THIS TIME THE PARTICIPANT WILL NOT SHOW YOU WHICH PIECE SHE/HE HAS TAKEN]

1. Did you consume this food in your household in the last **dry season/summer** (between June 2021, when the river level started to decrease, and November 2021, when the river was low)?

( ) Yes ( ) No

[IF THE ANSWER IS **NO, go to question B**]

In the last dry season/summer (between June 2021, when the river started to flow, and November 2021, when the river was dry), you did this: every week, every month or less than every month?

| ( ) Every week | How many kg of this food did you eat per week in the last dry season? __________ kg |
| --- | --- |
| ( ) Every month | How many kg of this food did you eat per month in the last dry season? __________ kg |
| ( ) Less than every month | How many kg of this food did you eat in the last dry season? __________ kg |

1. Did you consume this food in your household in the last **wet season/winter** (between December 2021, when the river level started to increase, and May 2022, when the river was high)?

( ) Yes ( ) No

[IF THE ANSWER IS **NO** to **A** and **B** and if the participant doesn`t get the method, repeat the example with beef and/or soy]

In the last wet season/winter (between December 2021, when the river started to flood, and May 2022, when the river was flooded), you did this: every week, every month or less than every month?

| ( ) Every week | How many kg of this food did you eat per week in the last dry season? __________ kg |
| --- | --- |
| ( ) Every month | How many kg of this food did you eat per month in the last dry season? __________ kg |
| ( ) Less than every month | How many kg of this food did you eat in the last dry season? __________ kg |

[REVERSE THE ROLES, HAND THE BAG TO THE PARTICIPANT AND ASK THE PARTICIPANT TO ASK YOU THE FIRST QUESTION (LETTER A):

*You're going to ask if at my house we consumed this food in the last dry season/summer.* [WAIT FOR THE PARTICIPANT TO ASK THE QUESTION AND ANSWER:] *“Yes. At home, we consumed this food every month in the last dry season. That resulted, more or less, 2kg of this food per month in the last dry season”.*

***Do you know what food I'm talking about?*** *(****if yes****) How do you know this, or are you taking a guess? Have you seen my domino piece? So you don't know, do you? (****if not****) See how the domino won't let me know what food item you're talking about?*

*That was just an example. When I ask you the questions, you won't let me see the dominoes. So I will* ***NOT*** *know which item you are answering about. But when we put together all the interviews of all the people, we'll be able to calculate the percentage of people who eat chicken, for example. But we won't know who eats it and who doesn’t.*

**[DO NOT PROCEED** UNTIL YOU ARE SURE THE PARTICIPANT UNDERSTOOD HOW IT WORKS. IF NECESSARY, USE BEEF AND SOY EXAMPLES TO EXPLAIN MORE]

***Now that you understand how it works, I'm going to ask you more questions that will also be about the dry season (summer) and wet season (winter). We're going to draw the dominoes 8 times, okay? Let's start?***

| **Part 1.1 - Indirect questions - CONSUMPTION (PASTA OR MAMMALS AND BIRDS)** |
| --- |

1. *Here are two more items. Don't answer yet, wait to get the domino first.*

| **Item 1: eat pasta (packages)** | [SHOW BOTH ITEMS] |
| --- | --- |
| **Item 2: eat wild animals (wild meat)** |  |

*Shake the bag and get a domino piece. Don't let me see the result. Look at the result and remember it. If it is 1 (1 ball), answer the questions about the food item 1 (pasta), if it is 2 (2 balls), answer the questions about the food item 2 (wild meat). Do you know which item you will answer about? Okay, so I'll ask you:*

1. Did you consume this food item in your household in the last **dry season/summer** (between June 2021, when the river level started to decrease, and November 2021, when the river was low)?

( ) Yes ( ) No

[IF THE ANSWER IS **YES,** go to question **C**]

1. Have you ever consumed this food item in any dry season/summer in the past:

( ) Yes. [IF YES] When did you consume it the last time during the dry season (year): _______

( ) No

[IF IT WAS **LESS THAN 5 YEARS AGO** (BETWEEN 2021 AND 2017), GO TO QUESTION **D**].

[IF IT WAS **MORE THAN 5 YEARS AGO** (2016 AND BEFORE), GO TO QUESTION **E**]

[IF THE ANSWER IS **NO**, GO TO QUESTION **E**]

1. [IMPORTANT TO SAY TO THE PARTICIPANTS]: *I'm going to ask questions about the amount you’ve consumed of this food item. If you got the pasta, think about how many packages of pasta you’ve consumed. If you got wild meat, think about how many kg of wild meat you’ve consumed. You just need to tell me how many packages or kg you've consumed (eg 1, 1.5, 2). OK?*

In the last **dry season/summer** (between June 2021, when the river level started to decrease, and November 2021, when the river was low), you consumed this food item: every week, every month or less than every month?

| ( ) Every week | How many packages or kg did you consume per week in the last dry season? __________ packages/kg |
| --- | --- |
| ( ) Every month | How many packages or kg did you consume per month in the last dry season? __________ packages/kg |
| ( ) Less than every month | How many packages or kg did you consume in the last dry season? __________ packages/kg |

[IF **ANSWERED QUESTION C**, GO TO QUESTION **E**]

1. [IMPORTANT TO SAY TO THE PARTICIPANTS]: *I'm going to ask questions about the amount you’ve consumed of this food item. If you got the pasta, think about how many packages of pasta you’ve consumed. If you got wild meat, think about how many kg of wild meat you’ve consumed. You just need to tell me how many packages or kg you've consumed (eg 1, 1.5, 2). OK?*

The last time you consumed this food item during the **dry season/summer** (between June, when the river level started to decrease, and November, when the river was low), you did this: every week, every month or less than every month?

| ( ) Every week | How many packages or kg did you consume per week in the last time in the dry season? __________ packages/kg |
| --- | --- |
| ( ) Every month | How many packages or kg did you consume per month in the last time in the dry season? __________ packages/kg |
| ( ) Less than every month | How many packages or kg did you consume last time in the dry season? __________ packages/kg |

1. Did you consume this food item in your household in the last **wet season/winter** (between December 2021, when the river started to flood, and May 2022, when the river was flooded)?

( ) Yes ( ) No

[IF THE ANSWER IS **YES,** go to question **G**]

1. Have you ever consumed this food item in any wet season/winter in the past:

( ) Yes. [IF YES] When did you consume this food item the last time during the wet season (year): ___________

( ) No

[IF IT WAS **LESS THAN 5 YEARS AGO** (BETWEEN 2021 AND 2017), GO TO QUESTION **H**].

[IF IT WAS **MORE THAN 5 YEARS AGO** (2016 AND BEFORE), GO TO QUESTION **2**]

[IF THE ANSWER IS **NO**, GO TO QUESTION **2**]

1. [IMPORTANT TO SAY TO THE PARTICIPANTS]: *I'm going to ask questions about the amount you’ve consumed of this food item. If you got the pasta, think about how many packages of pasta you’ve consumed. If you got wild meat, think about how many kg of wild meat you’ve consumed. You just need to tell me how many packages or kg you've consumed (eg 1, 1.5, 2). OK?*

In the last **wet season/winter** (between December 2021, when the river started to flood, and May 2022, when the river was flooded), you consumed this food item: every week, every month or less than every month?

| ( ) Every week | How many packages or kg did you consume per week in the last wet season? __________ packages/kg |
| --- | --- |
| ( ) Every month | How many packages or kg did you consume per month in the last wet season? __________ packages/kg |
| ( ) Less than every month | How many packages or kg did you consume in the last wet season? __________ packages/kg |

[IF **ANSWERED QUESTION G**, GO TO QUESTION **2**]

1. [IMPORTANT TO SAY TO THE PARTICIPANTS]: *I'm going to ask questions about the amount you’ve consumed of this food item. If you got the pasta, think about how many packages of pasta you’ve consumed. If you got wild meat, think about how many kg of wild meat you’ve consumed. You just need to tell me how many packages or kg you've consumed (eg 1, 1.5, 2). OK?*

The last time you consumed this food item during the **wet season/winter** (between December, when the river started to flood, and May, when the river was flooded), you did this: every week, every month or less than every month?

| ( ) Every week | How many packages or kg did you consume per week in the last time in the wet season? __________ packages/kg |
| --- | --- |
| ( ) Every month | How many packages or kg did you consume per month in the last time in the wet season? __________ packages/kg |
| ( ) Less than every month | How many packages or kg did you consume last time in the wet season? __________ packages/kg |

| **Part 1.2 - Indirect questions - CONSUMPTION (CORN DISH OR CHELONIANS)** |
| --- |

1. *Here are two more items. Don't answer yet, wait to get the domino first.*

| **Item 1: to eat corn dish** | [SHOW BOTH ITEMS] |
| --- | --- |
| **Item 2: to eat chelonians** |  |

*Shake the bag and get a domino piece. Don't let me see the result. Look at the result and remember it. If it is 1 (1 ball), answer the questions about the food item 1 (corn dish), if it is 2 (2 balls), answer the questions about the food item 2 (chelonians). Do you know which item you will answer about? Okay, so I'll ask you:*

1. Did you consume this food item in your household in the last **dry season/summer** (between June 2021, when the river level started to decrease, and November 2021, when the river was low)?

( ) Yes ( ) No

[IF THE ANSWER IS **YES,** go to question **C**]

1. Have you ever consumed this food item in any dry season/summer in the past:

( ) Yes. [IF YES] When did you consume it the last time during the dry season (year): _______

( ) No

[IF IT WAS **LESS THAN 5 YEARS AGO** (BETWEEN 2021 AND 2017), GO TO QUESTION **D**].

[IF IT WAS **MORE THAN 5 YEARS AGO** (2016 AND BEFORE), GO TO QUESTION **E**]

[IF THE ANSWER IS **NO**, GO TO QUESTION **E**]

1. [IMPORTANT TO SAY TO THE PARTICIPANTS]: *I'm going to ask questions about how many of this food item you consumed. If you got the corn dish, think about how many corn dishes you’ve consumed. If you got chelonians, think about how many chelonians you’ve consumed. You just need to tell me how many corn dishes or chelonians you've consumed (eg 1, 2, 3). OK?*

In the last **dry season/summer** (between June 2021, when the river level started to decrease, and November 2021, when the river was low), you consumed this food item: every week, every month or less than every month?

| ( ) Every week | How many units did you consume per week in the last dry season? __________ units |
| --- | --- |
| ( ) Every month | How many units did you consume per month in the last dry season? __________ units |
| ( ) Less than every month | How many units did you consume in the last dry season? __________ units |

[IF **ANSWERED QUESTION C**, GO TO QUESTION **E**]

1. [IMPORTANT TO SAY TO THE PARTICIPANTS]: *I'm going to ask questions about how many of this food item you consumed. If you got the corn dish, think about how many corn dishes you’ve consumed. If you got chelonians, think about how many chelonians you’ve consumed. You just need to tell me how many corn dishes or chelonians you've consumed (eg 1, 2, 3). OK?*

The last time you consumed this food item during the **dry season/summer** (between June, when the river level started to decrease, and November, when the river was low), you did this: every week, every month or less than every month?

| ( ) Every week | How many units did you consume per week in the last time in the dry season? __________ units |
| --- | --- |
| ( ) Every month | How many units did you consume per month in the last time in the dry season? __________ units |
| ( ) Less than every month | How many units did you consume last time in the dry season? __________ units |

1. Did you consume this food item in your household in the last **wet season/winter** (between December 2021, when the river started to flood, and May 2022, when the river was flooded)?

( ) Yes ( ) No

[IF THE ANSWER IS **YES,** go to question **G**]

1. Have you ever consumed this food item in any wet season/winter in the past:

( ) Yes. [IF YES] When did you consume this food item the last time during the wet season (year): ___________

( ) No

[IF IT WAS **LESS THAN 5 YEARS AGO** (BETWEEN 2021 AND 2017), GO TO QUESTION **H**].

[IF IT WAS **MORE THAN 5 YEARS AGO** (2016 AND BEFORE), GO TO QUESTION **3**]

[IF THE ANSWER IS **NO**, GO TO QUESTION **3**]

1. [IMPORTANT TO SAY TO THE PARTICIPANTS]: *I'm going to ask questions about how many of this food item you consumed. If you got the corn dish, think about how many corn dishes you’ve consumed. If you got chelonians, think about how many chelonians you’ve consumed. You just need to tell me how many corn dishes or chelonians you've consumed (eg 1, 2, 3). OK?*

In the last **wet season/winter** (between December 2021, when the river started to flood, and May 2022, when the river was flooded), you consumed this food item: every week, every month or less than every month?

| ( ) Every week | How many units did you consume per week in the last wet season? __________ units |
| --- | --- |
| ( ) Every month | How many units did you consume per month in the last wet season? __________ units |
| ( ) Less than every month | How many units did you consume in the last wet season? __________ units |

[IF **ANSWERED QUESTION G**, GO TO QUESTION **3**]

1. [IMPORTANT TO SAY TO THE PARTICIPANTS]: *I'm going to ask questions about how many of this food item you consumed. If you got the corn dish, think about how many corn dishes you’ve consumed. If you got chelonians, think about how many chelonians you’ve consumed. You just need to tell me how many corn dishes or chelonians you've consumed (eg 1, 2, 3). OK?*

The last time you consumed this food item during the **wet season/winter** (between December, when the river started to flood, and May, when the river was flooded), you did this: every week, every month or less than every month?

| ( ) Every week | How many units did you consume per week in the last time in the wet season? __________ units |
| --- | --- |
| ( ) Every month | How many units did you consume per month in the last time in the wet season? __________ units |
| ( ) Less than every month | How many units did you consume last time in the wet season? __________ units |

| **Part 1.3 - Indirect questions - Barter (EAT CASSAVA OR TRADE WILD MEAT)** |
| --- |

1. *Here are two more items. Don't answer yet, wait to get the domino first.*

| **Item 1: eat cassava** | [SHOW BOTH ITEMS] |
| --- | --- |
| **Item 2: Barter wild meat. For example when you give wild meat as a gift or exchange it and receive anything in return** |  |

*Shake the bag and get a domino piece. Don't let me see the result. Look at the result and remember it. If it is 1 (1 ball), answer the questions about item 1 (eating cassava), if it is 2 (2 balls), answer the questions about item 2 (barter trading wild meat). Do you know which item you will answer about? Okay, so I'll ask you:*

1. Did you do this in your household in the last **dry season/summer** (between June 2021, when the river level started to decrease, and November 2021, when the river was low)?

( ) Yes ( ) No

[IF THE ANSWER IS **YES,** go to question **C**]

1. Have you ever done this in any dry season/summer in the past:

( ) Yes. [IF YES] When did you do it the last time during the dry season (year): ____________

( ) No

[IF IT WAS **LESS THAN 5 YEARS AGO** (BETWEEN 2021 AND 2017), GO TO QUESTION **D**].

[IF IT WAS **MORE THAN 5 YEARS AGO** (2016 AND BEFORE), GO TO QUESTION **E**]

[IF THE ANSWER IS **NO**, GO TO QUESTION **E**]

1. [IMPORTANT TO SAY TO THE PARTICIPANTS]: *I'm going to ask questions about how much you've done this. If you got cassava, think about how many kg of cassava you ate. If you got wild meat, think about how many kg of wild meat you bartered. You just need to tell me how many kg you ate or bartered (eg 1, 1.5, 2). OK?*

In the last **dry season/summer** (between June 2021, when the river level started to decrease, and November 2021, when the river was low), you did this: every week, every month or less than every month?

| ( ) Every week | How many kg did you eat or trade per week in the last dry season? __________ kg |
| --- | --- |
| ( ) Every month | How many kg did you eat or trade per month in the last dry season? __________ kg |
| ( ) Less than every month | How many kg did you eat or trade in the last dry season? __________ kg |

[IF **ANSWERED QUESTION C**, GO TO QUESTION **E**]

1. [IMPORTANT TO SAY TO THE PARTICIPANTS]: *I'm going to ask questions about how much you've done this. If you got cassava, think about how many kg of cassava you ate. If you got wild meat, think about how many kg of wild meat you bartered. You just need to tell me how many kg you ate or bartered (eg 1, 1.5, 2). OK?*

In the last time you did this during the **dry season/summer** (between June, when the river level started to decrease, and November, when the river was low), you did this: every week, every month or less than every month?

| ( ) Every week | How many kg did you eat or trade per week in the last time in the dry season? __________ kg |
| --- | --- |
| ( ) Every month | How many kg did you eat or trade per month in the last time in the dry season? __________ kg |
| ( ) Less than every month | How many kg did you eat or trade last time in the dry season? __________ kg |

1. Did you do this in your household in the last **wet season/winter** (between December 2021, when the river started to flood, and May 2022, when the river was flooded)?

( ) Yes ( ) No

[IF THE ANSWER IS **YES,** go to question **G**]

1. Have you ever done this in any wet season/winter in the past:

( ) Yes. [IF YES] When did you do it the last time during the wet season (year): ____________

( ) No

[IF IT WAS **LESS THAN 5 YEARS AGO** (BETWEEN 2021 AND 2017), GO TO QUESTION **H**].

[IF IT WAS **MORE THAN 5 YEARS AGO** (2016 AND BEFORE), GO TO QUESTION **4**]

[IF THE ANSWER IS **NO**, GO TO QUESTION **4**]

1. [IMPORTANT TO SAY TO THE PARTICIPANTS]: *I'm going to ask questions about how much you've done this. If you got cassava, think about how many kg of cassava you ate. If you got wild meat, think about how many kg of wild meat you bartered. You just need to tell me how many kg you ate or bartered (eg 1, 1.5, 2). OK?*

In the last **wet season/winter** (between December 2021, when the river started to flood, and May 2022, when the river was flooded), you did this: every week, every month or less than every month?

| ( ) Every week | How many kg did you eat or trade per week in the last wet season? __________ kg |
| --- | --- |
| ( ) Every month | How many kg did you eat or trade per month in the last wet season? __________ kg |
| ( ) Less than every month | How many kg did you eat or trade in the last wet season? __________ kg |

[IF **ANSWERED QUESTION G**, GO TO QUESTION **4**]

1. [IMPORTANT TO SAY TO THE PARTICIPANTS]: *I'm going to ask questions about how much you've done this. If you got cassava, think about how many kg of cassava you ate. If you got wild meat, think about how many kg of wild meat you bartered. You just need to tell me how many kg you ate or bartered (eg 1, 1.5, 2). OK?*

In the last time you did this during the **wet season/winter** (between December, when the river started to flood, and May, when the river was flooded), you did this: every week, every month or less than every month?

| ( ) Every week | How many kg did you eat or trade per week in the last time in the wet season? __________ kg |
| --- | --- |
| ( ) Every month | How many kg did you eat or trade per month in the last time in the wet season? __________ kg |
| ( ) Less than every month | How many kg did you eat or trade last time in the wet season? __________ kg |

| **Part 1.4 - Indirect questions - Barter (BUY VEGETABLE OIL BOTTLES OR TRADE CHELONIANS)** |
| --- |

1. *Here are two more items. Don't answer yet, wait to get the domino first.*

| **Item 1: buy bottles of vegetable oil** | [SHOW BOTH ITEMS] |
| --- | --- |
| **Item 2: Barter chelonians. For example when you give a chelonian as a gift or exchange it and receive anything in return** |  |

*Shake the bag and get a domino piece. Don't let me see the result. Look at the result and remember it. If it is 1 (1 ball), answer the questions about item 1 (buying cooking oil), if it is 2 (2 balls), answer the questions about item 2 (barter trading chelonians). Do you know which item you will answer about? Okay, so I'll ask you:*

1. Did you do this in your household in the last **dry season/summer** (between June 2021, when the river level started to decrease, and November 2021, when the river was low)?

( ) Yes ( ) No

[IF THE ANSWER IS **YES,** go to question **C**]

1. Have you ever done this in any dry season/summer in the past:

( ) Yes. [IF YES] When did you do it the last time during the dry season (year): ____________

( ) No

[IF IT WAS **LESS THAN 5 YEARS AGO** (BETWEEN 2021 AND 2017), GO TO QUESTION **D**].

[IF IT WAS **MORE THAN 5 YEARS AGO** (2016 AND BEFORE), GO TO QUESTION **E**]

[IF THE ANSWER IS **NO**, GO TO QUESTION **E**]

1. [IMPORTANT TO SAY TO THE PARTICIPANTS]: *I'm going to ask questions about how much you've done this. If you got vegetable oil, think about how many bottles of vegetable oil you bought. If you got chelonians, think about how many chelonians you bartered. You just need to tell me how many bottles of vegetable oil you bought or chelonians you bartered (eg 1, 2, 3). OK?*

In the last **dry season/summer** (between June 2021, when the river started to flow, and November 2021, when the river was dry), you did this: every week, every month or less than every month?

| ( ) Every week | How many units did you buy or trade per week in the last dry season? __________ units |
| --- | --- |
| ( ) Every month | How many units did you buy or trade per month in the last dry season? __________ units |
| ( ) Less than every month | How many units did you buy or trade in the last dry season? __________ units |

[IF **ANSWERED QUESTION C**, GO TO QUESTION **E**]

1. [IMPORTANT TO SAY TO THE PARTICIPANTS]: *I'm going to ask questions about how much you've done this. If you got vegetable oil, think about how many bottles of vegetable oil you bought. If you got chelonians, think about how many chelonians you bartered. You just need to tell me how many bottles of vegetable oil you bought or chelonians you bartered (eg 1, 2, 3). OK?*

In the last time you did this during the **dry season/summer** (between June, when the river level started to decrease, and November, when the river was low), you did this: every week, every month or less than every month?

| ( ) Every week | How many units did you buy or trade per week in the last time in the dry season? __________ units |
| --- | --- |
| ( ) Every month | How many units did you buy or trade per month in the last time in the dry season? __________ units |
| ( ) Less than every month | How many units did you buy or trade last time in the dry season? __________ units |

1. Did you do this in your household in the last **wet season/winter** (between December 2021, when the river started to flood, and May 2022, when the river was flooded)?

( ) Yes ( ) No

[IF THE ANSWER IS **YES,** go to question **G**]

1. Have you ever done this in any wet season/winter in the past:

( ) Yes. [IF YES] When did you do it the last time during the wet season (year): ____________

( ) No

[IF IT WAS **LESS THAN 5 YEARS AGO** (BETWEEN 2021 AND 2017), GO TO QUESTION **H**].

[IF IT WAS **MORE THAN 5 YEARS AGO** (2016 AND BEFORE), GO TO QUESTION **5**]

[IF THE ANSWER IS **NO**, GO TO QUESTION **5**]

1. [IMPORTANT TO SAY TO THE PARTICIPANTS]: *I'm going to ask questions about how much you've done this. If you got vegetable oil, think about how many bottles of vegetable oil you bought. If you got chelonians, think about how many chelonians you bartered. You just need to tell me how many bottles of vegetable oil you bought or chelonians you bartered (eg 1, 2, 3). OK?*

In the last **wet season/winter** (between December 2021, when the river started to flood, and May 2022, when the river was flooded), you did this: every week, every month or less than every month?

| ( ) Every week | How many units did you buy or trade per week in the last wet season? __________ units |
| --- | --- |
| ( ) Every month | How many units did you buy or trade per month in the last wet season? __________ units |
| ( ) Less than every month | How many units did you buy or trade in the last wet season? __________ units |

[IF **ANSWERED QUESTION G**, GO TO QUESTION **5**]

1. [IMPORTANT TO SAY TO THE PARTICIPANTS]: *I'm going to ask questions about how much you've done this. If you got vegetable oil, think about how many bottles of vegetable oil you bought. If you got chelonians, think about how many chelonians you bartered. You just need to tell me how many bottles of vegetable oil you bought or chelonians you barteed (eg 1, 2, 3). OK?*

In the last time you did this during the **wet season/winter** (between December, when the river started to flood, and May, when the river was flooded), you did this: every week, every month or less than every month?

| ( ) Every week | How many units did you buy or trade per week in the last time in the wet season? __________ units |
| --- | --- |
| ( ) Every month | How many units did you buy or trade per month in the last time in the wet season? __________ units |
| ( ) Less than every month | How many units did you buy or trade last time in the wet season? __________ units |

| **Part 1.5 - Indirect questions - Trade (BUY BATTERIES OR TRADE WILD ANIMALS)** |
| --- |

1. *Here are two more items. Don't answer yet, wait to get the domino first.*

| **Item 1: buy batteries** | [SHOW BOTH ITEMS] |
| --- | --- |
| **Item 2: Trade wild animals** |  |

*Shake the bag and get a domino piece. Don't let me see the result. Look at the result and remember it. If it is 1 (1 ball), answer the questions about item 1 (buying batteries), if it is 2 (2 balls), answer the questions about item 2 (trading chelonians). Do you know which item you will answer about? Okay, so I'll ask you:*

1. Did you do this in your household in the last **dry season/summer** (between June 2021, when the river level started to decrease, and November 2021, when the river was low)?

( ) Yes ( ) No

[IF THE ANSWER IS **YES,** go to question **C**]

1. Have you ever done this in any dry season/summer in the past:

( ) Yes. [IF YES] When did you do it the last time during the dry season (year): ____________

( ) No

[IF IT WAS **LESS THAN 5 YEARS AGO** (BETWEEN 2021 AND 2017), GO TO QUESTION **D**].

[IF IT WAS **MORE THAN 5 YEARS AGO** (2016 AND BEFORE), GO TO QUESTION **E**]

[IF THE ANSWER IS **NO**, GO TO QUESTION **E**]

1. [IMPORTANT TO SAY TO THE PARTICIPANTS]: *I'm going to ask questions about how much you've done this. If you got batteries, think about how many units of batteries you bought. If you got wildlife, think about how many wild animals you traded. You just need to tell me how many batteries you bought or wild animals you traded (eg 1, 2, 3). OK?*

In the last **dry season/summer** (between June 2021, when the river level started to decrease, and November 2021, when the river was low), you did this: every week, every month or less than every month?

| ( ) Every week | How many units did you buy or trade per week in the last dry season? __________ units |
| --- | --- |
| ( ) Every month | How many units did you buy or trade per month in the last dry season? __________ units |
| ( ) Less than every month | How many units did you buy or trade in the last dry season? __________ units |

[IF **ANSWERED QUESTION C**, GO TO QUESTION **E**]

1. [IMPORTANT TO SAY TO THE PARTICIPANTS]: *I'm going to ask questions about how much you've done this. If you got batteries, think about how many units of batteries you bought. If you got wildlife, think about how many wild animals you traded. You just need to tell me how many batteries you bought or wild animals you traded (eg 1, 2, 3). OK?*

In the last time you did this during the **dry season/summer** (between June, when the river level started to decrease, and November, when the river was low), you did this: every week, every month or less than every month?

| ( ) Every week | How many units did you buy or trade per week in the last time in the dry season? __________ units |
| --- | --- |
| ( ) Every month | How many units did you buy or trade per month in the last time in the dry season? __________ units |
| ( ) Less than every month | How many units did you buy or trade last time in the dry season? __________ units |

1. Did you do this in your household in the last **wet season/winter** (between December 2021, when the river started to flood, and May 2022, when the river was flooded)?

( ) Yes ( ) No

[IF THE ANSWER IS **YES,** go to question **G**]

1. Have you ever done this in any wet season/winter in the past:

( ) Yes. [IF YES] When did you do it the last time during the wet season (year): ____________

( ) No

[IF IT WAS **LESS THAN 5 YEARS AGO** (BETWEEN 2021 AND 2017), GO TO QUESTION **H**].

[IF IT WAS **MORE THAN 5 YEARS AGO** (2016 AND BEFORE), GO TO QUESTION **6**]

[IF THE ANSWER IS **NO**, GO TO QUESTION **6**]

1. [IMPORTANT TO SAY TO THE PARTICIPANTS]: *I'm going to ask questions about how much you've done this. If you got batteries, think about how many units of batteries you bought. If you got wildlife, think about how many wild animals you traded. You just need to tell me how many batteries you bought or wild animals you traded (eg 1, 2, 3). OK?*

In the last **wet season/winter** (between December 2021, when the river started to flood, and May 2022, when the river was flooded), you did this: every week, every month or less than every month?

| ( ) Every week | How many units did you buy or trade per week in the last wet season? __________ units |
| --- | --- |
| ( ) Every month | How many units did you buy or trade per month in the last wet season? __________ units |
| ( ) Less than every month | How many units did you buy or trade in the last wet season? __________ units |

[IF **ANSWERED QUESTION G**, GO TO QUESTION **6**]

1. [IMPORTANT TO SAY TO THE PARTICIPANTS]: *I'm going to ask questions about how much you've done this. If you got batteries, think about how many units of batteries you bought. If you got wildlife, think about how many wild animals you traded. You just need to tell me how many batteries you bought or wild animals you traded (eg 1, 2, 3). OK?*

In the last time you did this during the **wet season/winter** (between December, when the river started to flood, and May, when the river was flooded), you did this: every week, every month or less than every month?

| ( ) Every week | How many units did you buy or trade per week in the last time in the wet season? __________ units |
| --- | --- |
| ( ) Every month | How many units did you buy or trade per month in the last time in the wet season? __________ units |
| ( ) Less than every month | How many units did you buy or trade last time in the wet season? __________ units |

| **Part 1.6 - Indirect questions - Trade (BUY TOOTHBRUSHES OR TRADE CHELONIANS)** |
| --- |

1. *Here are two more items. Don't answer yet, wait to get the domino first.*

| **Item 1: buy toothbrushes** | [SHOW BOTH ITEMS] |
| --- | --- |
| **Item 2: Trade chelonians** |  |

*Shake the bag and get a domino piece. Don't let me see the result. Look at the result and remember it. If it is 1 (1 ball), answer the questions about item 1 (buying toothbrushes), if it is 2 (2 balls), answer the questions about item 2 (trading chelonians). Do you know which item you will answer about? Okay, so I'll ask you:*

1. Did you do this in your household in the last **dry season/summer** (between June 2021, when the river level started to decrease, and November 2021, when the river was low)?

( ) Yes ( ) No

[IF THE ANSWER IS **YES,** go to question **C**]

1. Have you ever done this in any dry season/summer in the past:

( ) Yes. [IF YES] When did you do it the last time during the dry season (year): ____________

( ) No

[IF IT WAS **LESS THAN 5 YEARS AGO** (BETWEEN 2021 AND 2017), GO TO QUESTION **D**].

[IF IT WAS **MORE THAN 5 YEARS AGO** (2016 AND BEFORE), GO TO QUESTION **E**]

[IF THE ANSWER IS **NO**, GO TO QUESTION **E**]

1. [IMPORTANT TO SAY TO THE PARTICIPANTS]: *I'm going to ask questions about how much you've done this. If you got toothbrushes, think about how many units of toothbrushes you bought. If you got chelonians, think about how many chelonians you traded. You just need to tell me how many toothbrushes you bought or chelonians you traded (eg 1, 2, 3). OK?*

In the last **dry season/summer** (between June 2021, when the river level started to decrease, and November 2021, when the river was low), you did this: every week, every month or less than every month?

| ( ) Every week | How many units did you buy or trade per week in the last dry season? __________ units |
| --- | --- |
| ( ) Every month | How many units did you buy or trade per month in the last dry season? __________ units |
| ( ) Less than every month | How many units did you buy or trade in the last dry season? __________ units |

[IF **ANSWERED QUESTION C**, GO TO QUESTION **E**]

1. [IMPORTANT TO SAY TO THE PARTICIPANTS]: *I'm going to ask questions about how much you've done this. If you got toothbrushes, think about how many units of toothbrushes you bought. If you got chelonians, think about how many chelonians you traded. You just need to tell me how many toothbrushes you bought or chelonians you traded (eg 1, 2, 3). OK?*

In the last time you did this during the **dry season/summer** (between June, when the river level started to decrease, and November, when the river was low), you did this: every week, every month or less than every month?

| ( ) Every week | How many units did you buy or trade per week in the last time in the dry season? __________ units |
| --- | --- |
| ( ) Every month | How many units did you buy or trade per month in the last time in the dry season? __________ units |
| ( ) Less than every month | How many units did you buy or trade last time in the dry season? __________ units |

1. Did you do this in your household in the last **wet season/winter** (between December 2021, when the river started to flood, and May 2022, when the river was flooded)?

( ) Yes ( ) No

[IF THE ANSWER IS **YES,** go to question **G**]

1. Have you ever done this in any wet season/winter in the past:

( ) Yes. [IF YES] When did you do it the last time during the wet season (year): ____________

( ) No

[IF IT WAS **LESS THAN 5 YEARS AGO** (BETWEEN 2021 AND 2017), GO TO QUESTION **H**].

[IF IT WAS **MORE THAN 5 YEARS AGO** (2016 AND BEFORE), GO TO PART 2]

[IF THE ANSWER IS **NO**, GO TO PART 2]

1. [IMPORTANT TO SAY TO THE PARTICIPANTS]: *I'm going to ask questions about how much you've done this. If you got toothbrushes, think about how many units of toothbrushes you bought. If you got chelonians, think about how many chelonians you traded. You just need to tell me how many toothbrushes you bought or chelonians you traded (eg 1, 2, 3). OK?*

In the last **wet season/winter** (between December 2021, when the river started to flood, and May 2022, when the river was flooded), you did this: every week, every month or less than every month?

| ( ) Every week | How many units did you buy or trade per week in the last wet season? __________ units |
| --- | --- |
| ( ) Every month | How many units did you buy or trade per month in the last wet season? __________ units |
| ( ) Less than every month | How many units did you buy or trade in the last wet season? __________ units |

[IF **ANSWERED QUESTION G**, GO TO PART 2]

1. [IMPORTANT TO SAY TO THE PARTICIPANTS]: *I'm going to ask questions about how much you've done this. If you got toothbrushes, think about how many units of toothbrushes you bought. If you got chelonians, think about how many chelonians you traded. You just need to tell me how many toothbrushes you bought or chelonians you traded (eg 1, 2, 3). OK?*

In the last time you did this during the **wet season/winter** (between December, when the river started to flood, and May, when the river was flooded), you did this: every week, every month or less than every month?

| ( ) Every week | How many units did you buy or trade per week in the last time in the wet season? __________ units |
| --- | --- |
| ( ) Every month | How many units did you buy or trade per month in the last time in the wet season? __________ units |
| ( ) Less than every month | How many units did you buy or trade last time in the wet season? __________ units |

| **Part 2 - Social information** |
| --- |

*Finally, I'd like to ask you a few questions about you and your family. Often, what we have, eat, buy or exchange at home depends on our relationships, where we live or where we come from. So, this information will help us better understand how you get the products and food for your home. May I continue?*

| 9. What’s your age? _____________ | | And your main occupation? ______________________ | | |
| --- | --- | --- | --- | --- |
| ( ) Wage labor | ( ) Farmer | ( ) Fisher | ( ) Housewife | ( ) Retired |
| ( ) Trader | ( ) Entrepreneur | ( ) Sawyer | ( ) Not sure | ( ) Other______ |

10. Besides you, who else lives in this household? And what is their age and main occupation?

| **Relationship** | **How many?** | **What is(are) the age(s)?** | **What is(are) the main occupation(s)?** |
| --- | --- | --- | --- |
| Husband |  |  |  |
| Wife |  |  |  |
| Son |  |  |  |
| Daughter |  |  |  |
| Stepson |  |  |  |
| Stepdaughter |  |  |  |
| Grandson |  |  |  |
| Granddaughter |  |  |  |
| Father |  |  |  |
| Mother |  |  |  |
| Stepfather |  |  |  |
| Stepmother |  |  |  |
| Father in law |  |  |  |
| Mother in law |  |  |  |
| Son in law |  |  |  |
| Daughter in law |  |  |  |
| Brother in law |  |  |  |
| Sister in law |  |  |  |
| Uncle |  |  |  |
| Aunt |  |  |  |
| Cousin (male) |  |  |  |
| Cousin (female) |  |  |  |
| Nephew |  |  |  |
| Niece |  |  |  |
| Godson |  |  |  |
| Goddaughter |  |  |  |
| Friend (male) |  |  |  |
| Friend (female) |  |  |  |
| *Compadre* - son’s or daughter’s socially attributed father in the absence of birth father |  |  |  |
| *Comadre* - son’s or daughter’s socially attributed mother in the absence of birth mother |  |  |  |
| Brother |  |  |  |
| Sister |  |  |  |
| Other (male): ___________ |  |  |  |
| Other (female): _________ |  |  |  |

11. Do you have and/or maintain house/property in: [the rural area [outside the urban area, such as in a village, riverside, farm, by the road] / the urban area]? ( ) Yes ( ) No

12. How often do you or anyone else in the household go to the [rural area/urban center] during the **summer (dry season), which lasts from June to November**?

| ____ times per | ( ) day | ( ) week | ( ) month | ( ) dry season | ( ) never |
| --- | --- | --- | --- | --- | --- |

[IF THE HOUSEHOLD MEMBERS DOES NOT GO TO THE OTHER AREA, GO TO QUESTION 17]

1. How long do you or anyone else in the household spend in the [rural area/urban center] each time you go there during the **summer (dry season)**?

| ___________ | ( ) hours | ( ) days | ( ) weeks | ( ) months | ( ) dry season |
| --- | --- | --- | --- | --- | --- |

1. How far is this [rural area/urban area] from here during the summer and what is the main means of transportation used?

| Means of transportation 1: ________________________ | | | Distance (hours) 1: ______________ | |
| --- | --- | --- | --- | --- |
| ( ) Motorcycle | ( ) Bus | ( ) On foot | ( ) Big boat | ( ) Small boat |
| ( ) Car | ( ) Truck | ( ) Bicycle | ( ) Medium boat | ( ) Speedboat |
| ( ) Airplane | ( ) Canoe | ( ) Other: ____________________________________ | | |

| Means of transportation 2: ________________________ | | | Distance (hours) 2: ______________ | |
| --- | --- | --- | --- | --- |
| ( ) Motorcycle | ( ) Bus | ( ) On foot | ( ) Big boat | ( ) Small boat |
| ( ) Car | ( ) Truck | ( ) Bicycle | ( ) Medium boat | ( ) Speedboat |
| ( ) Airplane | ( ) Canoe | ( ) Other: ____________________________________ | | |

| Means of transportation 3: ________________________ | | | Distance (hours) 3: ______________ | |
| --- | --- | --- | --- | --- |
| ( ) Motorcycle | ( ) Bus | ( ) On foot | ( ) Big boat | ( ) Small boat |
| ( ) Car | ( ) Truck | ( ) Bicycle | ( ) Medium boat | ( ) Speedboat |
| ( ) Airplane | ( ) Canoe | ( ) Other: ____________________________________ | | |

13. How often do you or anyone else in the household go to the [rural area/urban center] during the **winter (wet season), which lasts from December to May**?

| ____ times per | ( ) day | ( ) week | ( ) month | ( ) dry season | ( ) never |
| --- | --- | --- | --- | --- | --- |

[IF THE HOUSEHOLD MEMBERS DOES NOT GO TO THE OTHER AREA, FINISH THE SURVEY]

1. How long do you or anyone else in the household spend in the [rural area/urban center] each time you go there during the **winter (wet season)**?

| ___________ | ( ) hours | ( ) days | ( ) weeks | ( ) months | ( ) dry season |
| --- | --- | --- | --- | --- | --- |

1. How far is this [rural area/urban area] from here during the winter and what is the main means of transportation used?

| Means of transportation 1: ________________________ | | | Distance (hours) 1: ______________ | |
| --- | --- | --- | --- | --- |
| ( ) Motorcycle | ( ) Bus | ( ) On foot | ( ) Big boat | ( ) Small boat |
| ( ) Car | ( ) Truck | ( ) Bicycle | ( ) Medium boat | ( ) Speedboat |
| ( ) Airplane | ( ) Canoe | ( ) Other: ____________________________________ | | |

| Means of transportation 2: ________________________ | | | Distance (hours) 2: ______________ | |
| --- | --- | --- | --- | --- |
| ( ) Motorcycle | ( ) Bus | ( ) On foot | ( ) Big boat | ( ) Small boat |
| ( ) Car | ( ) Truck | ( ) Bicycle | ( ) Medium boat | ( ) Speedboat |
| ( ) Airplane | ( ) Canoe | ( ) Other: ____________________________________ | | |

| Means of transportation 3: ________________________ | | | Distance (hours) 3: ______________ | |
| --- | --- | --- | --- | --- |
| ( ) Motorcycle | ( ) Bus | ( ) On foot | ( ) Big boat | ( ) Small boat |
| ( ) Car | ( ) Truck | ( ) Bicycle | ( ) Medium boat | ( ) Speedboat |
| ( ) Airplane | ( ) Canoe | ( ) Other: ____________________________________ | | |

**Appendix S3 –:** Images shown surveyed participants for indirect questions: Paired items and sensitive behavior

**
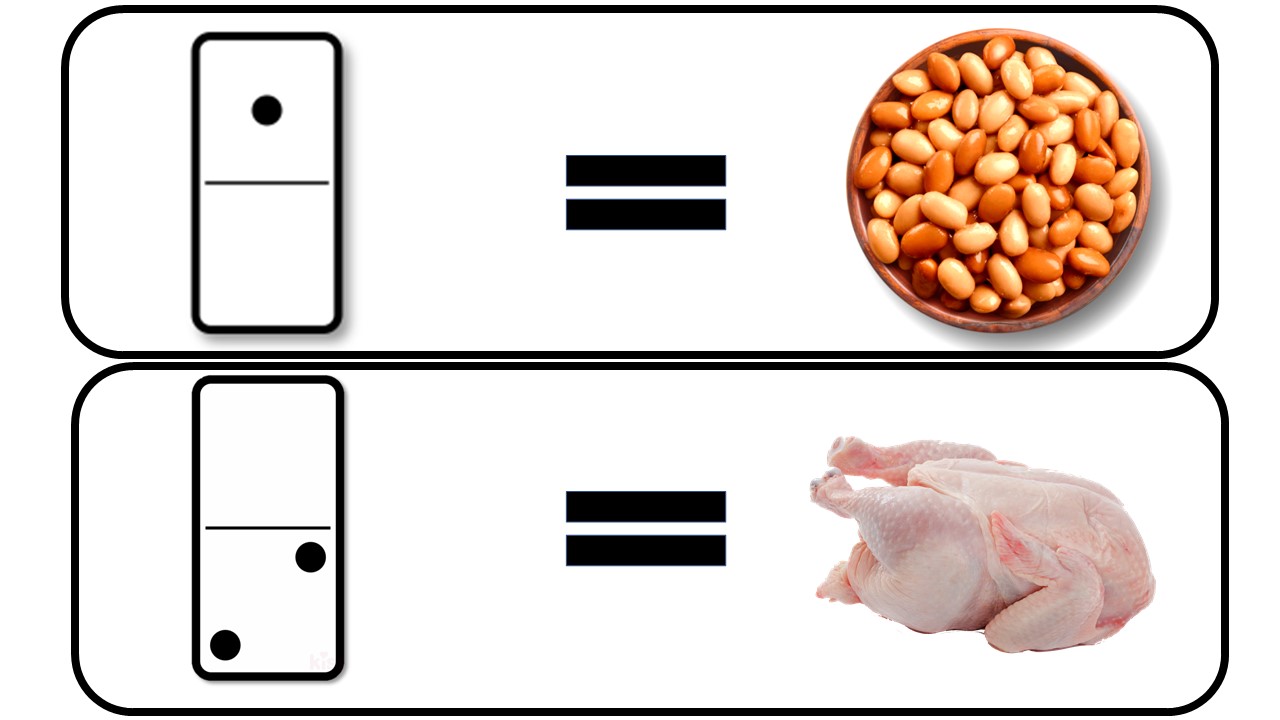
**

**
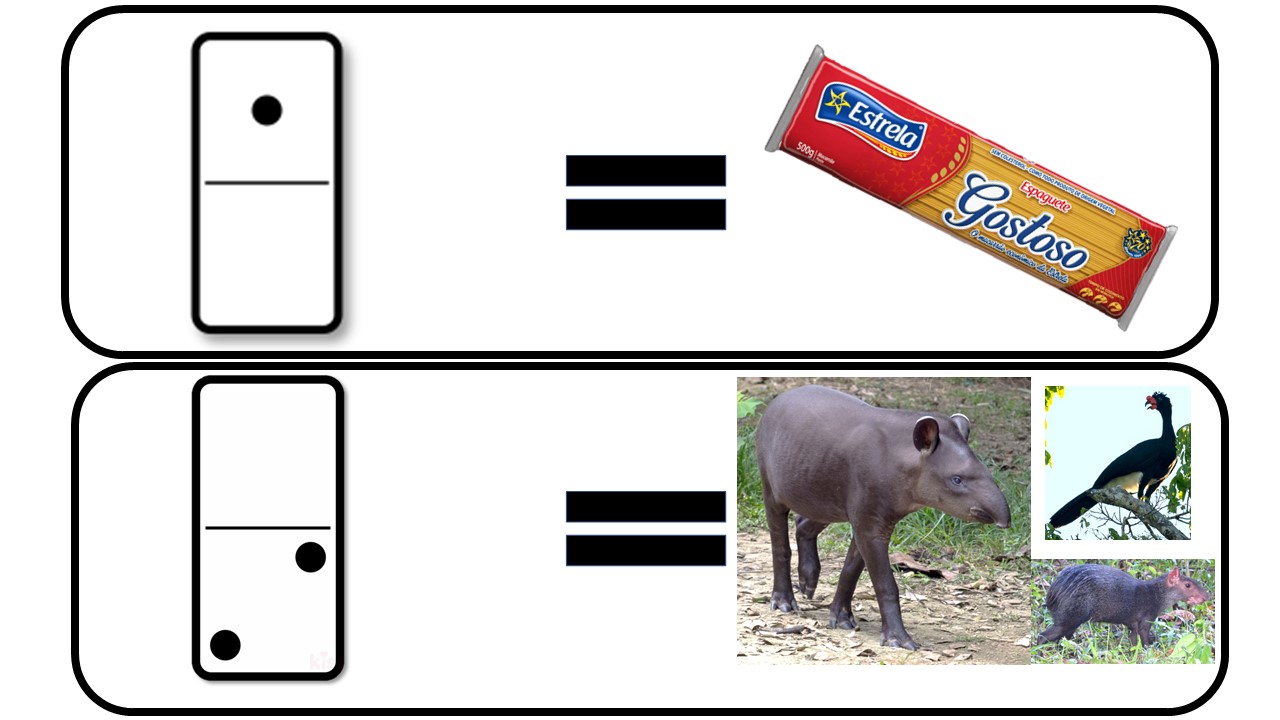
**

**
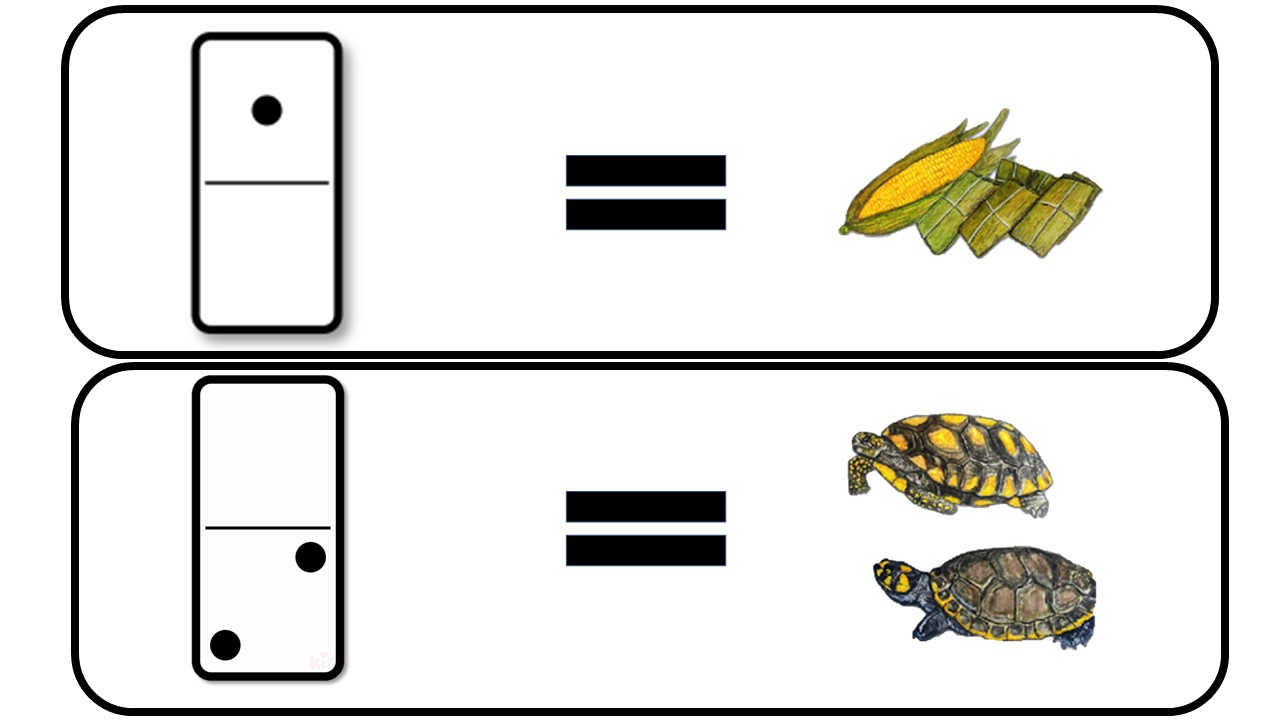
**

**
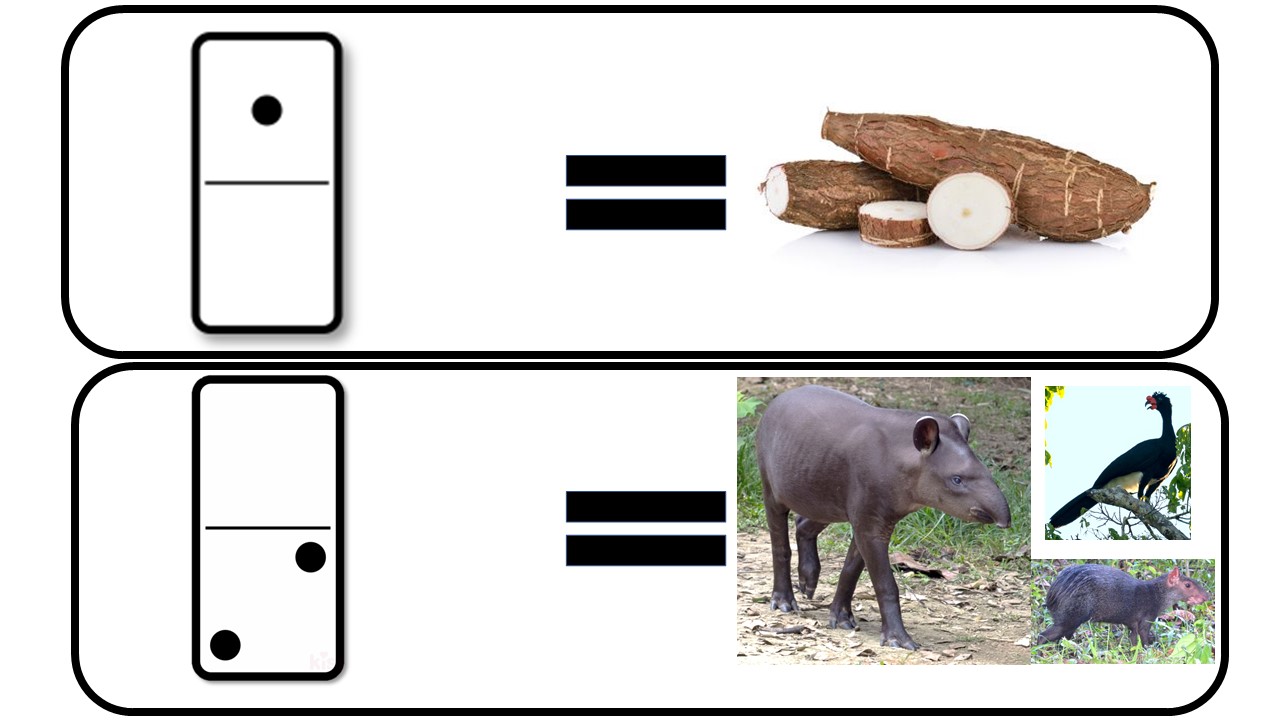
**

**
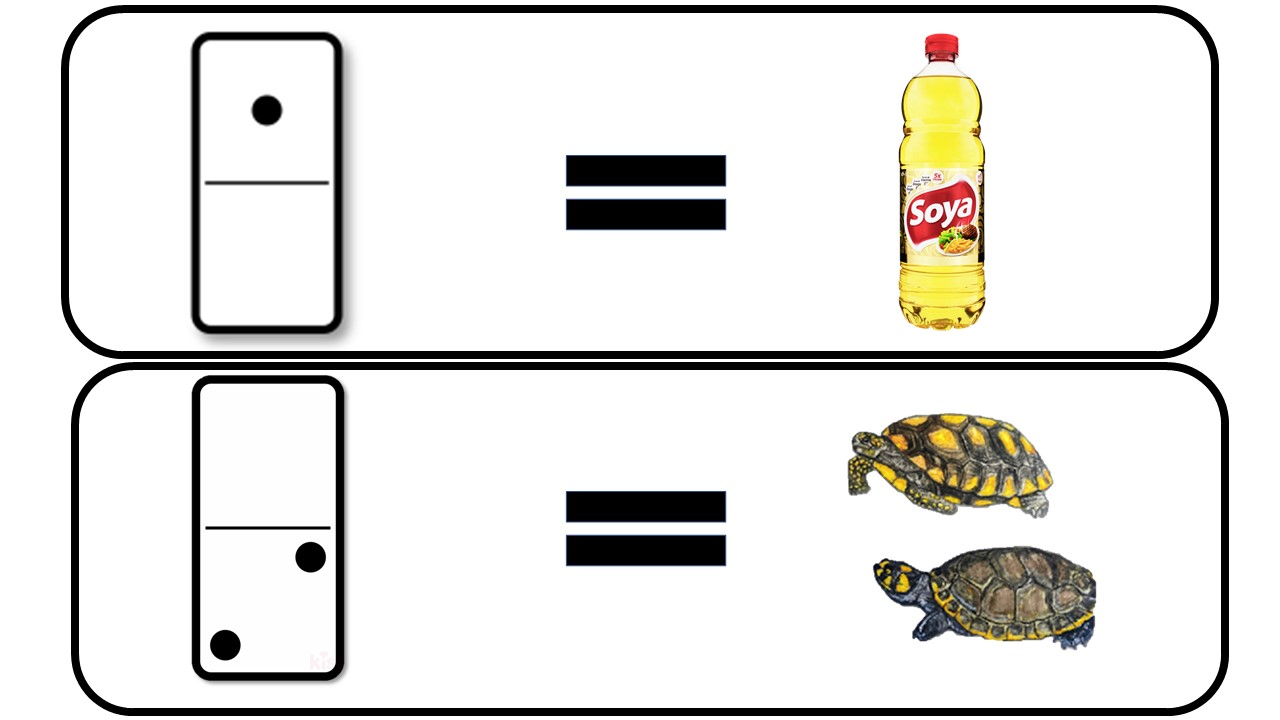
**

**
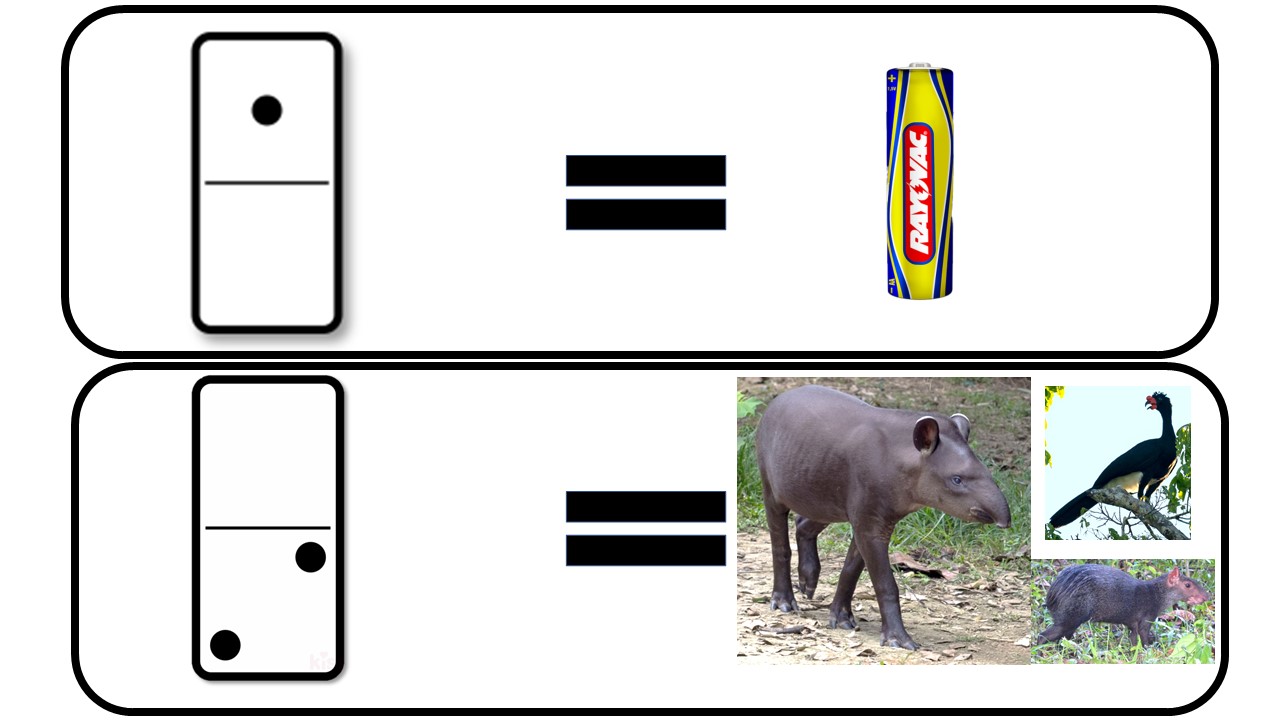
**

**
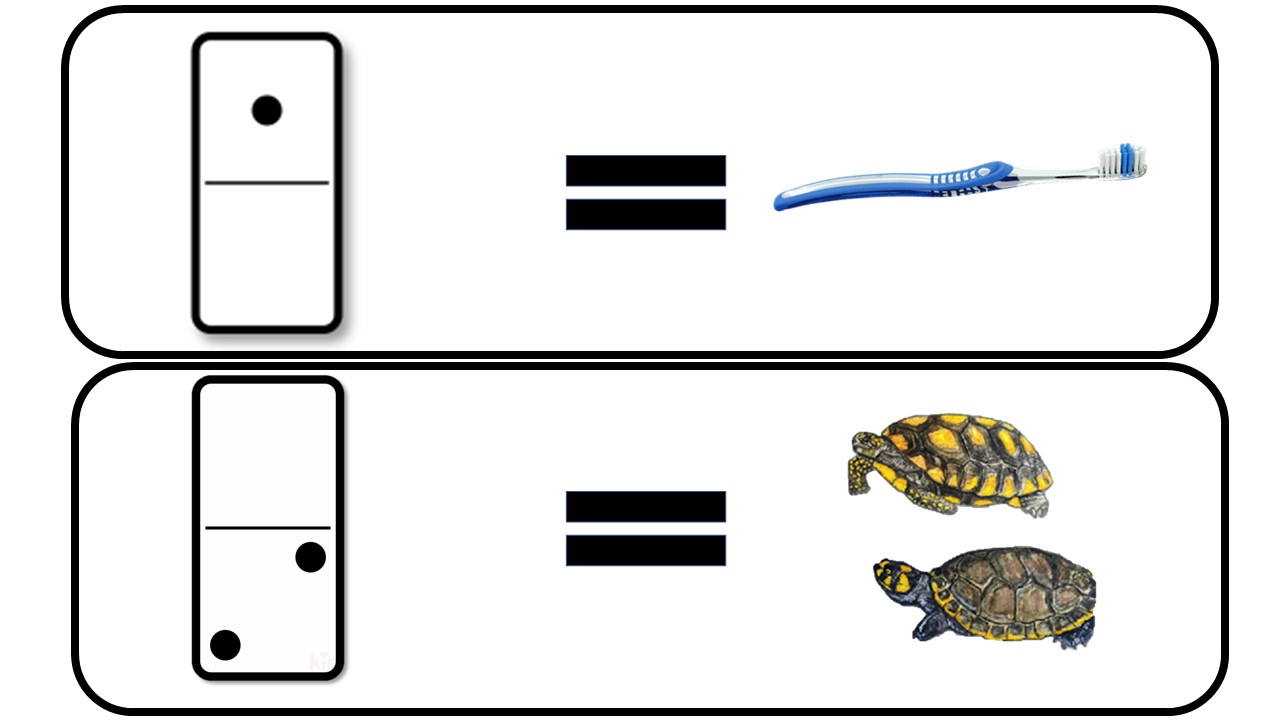
**

**Appendix S4 –.** Sample size per villages, districts and municipalities surveyed in this study according to major river basin, river margin, straight distance (in km) to reach the border of the urban area and number of households.

| **Municipality** | **Urbanization Category*** | **Village/  District ID** | **Major River Basin** | **River Margin** | **Straight Distance (km) to the border of urban area** | **Number of households** | **Number of surveys** |
| --- | --- | --- | --- | --- | --- | --- | --- |
| Manaus | Rural | 1 | Solimões | Left | 82 | 533 | 30 |
|  | Rural | 4 | Negro | Left | 59.9 | 67 | 30 |
|  | Rural | 5 | Negro | Left | 45.4 | 41 | 30 |
|  | Rural | 6 | Negro | Left | 41.9 | 92 | 30 |
|  | Peri-urban | 2 | Solimões | Right | 26 | 120 | 30 |
|  | Peri-urban | 7 | Negro | Left | 12 | 101 | 30 |
|  | Peri-urban | 3 | Solimões | Right | 2.9 | 107 | 30 |
|  | Urban | ZN | Negro | Left | 0 | - | 83 |
|  | Urban | ZL | Negro | Left | 0 | - | 72 |
|  | Urban | ZS | Negro | Left | 0 | - | 48 |
|  | Urban | ZO | Negro | Left | 0 | - | 42 |
|  | Urban | ZCS | Negro | Left | 0 | - | 30 |
|  | Urban | ZCO | Negro | Left | 0 | - | 24 |
| Carauari | Rural | 8 | Juruá | Left | 90.3 | 33 | 10 |
|  | Rural | 9 | Juruá | Left | 78 | 12 | 8 |
|  | Rural | 10 | Juruá | Right | 72.6 | 22 | 9 |
|  | Rural | 11 | Juruá | Right | 64 | 35 | 10 |
|  | Rural | 18 | Juruá | Right | 54.4 | 42 | 16 |
|  | Rural | 17 | Juruá | Right | 46.4 | 14 | 12 |
|  | Rural | 16 | Juruá | Right | 40 | 8 | 8 |
|  | Peri-urban | 12 | Juruá | Left | 39.5 | 119 | 10 |
|  | Peri-urban | 15 | Juruá | Left | 29 | 15 | 8 |
|  | Peri-urban | 14 | Juruá | Right | 21 | 15 | 10 |
|  | Peri-urban | 13 | Juruá | Right | 4.3 | 15 | 13 |
|  | Urban | CAF | Juruá | Left | 0 | - | 159 |

*Urban area refers to the urbanized area of a given municipality (more densely occupied and with higher infrastructure and build-up areas, IBGE 2010), the peri-urban area as the area ranging from the border of the urban area up to 40 km radius (on straight distance, or 12h by fluvial travel distance during the wet season), and rural area as any area from the border of the peri-urban area.

**Appendix S5 –.** Quantity categories per wildlife use and type of wild meat.

|  |  |  | **Categories of frequencies*** | | | |  | |
| --- | --- | --- | --- | --- | --- | --- | --- | --- |
| **Type of**  **wildlife use** | **Type of**  **Wild meat** | **Frequency**  **unit** | **1** | **2** | **3** | **4** | **5** | |
| Consumption | Mammals and birds | kg/month | 0 | 0 < x < 2.01 | 2.01 ≤ x < 4.03 | 4.03 ≤ x < 10.2 | | x ≥ 10.2 |
|  | Turtles and tortoises | units/month | 0 | 0 < x < 0.36 | 0.36 ≤ x < 1.11 | 1.11 ≤ x < 4.2 | | x ≥ 4.2 |
| Barter | Mammals and birds | kg/month | 0 | 0 < x < 0.42 | 0.42 ≤ x < 1.80 | 1.8 ≤ x < 5.1 | | x ≥ 5.1 |
|  | Turtles and tortoises | units/month | 0 | 0 < x < 1.5 | 1.5. ≤ x < 3.00 | 3 ≤ x < 4.29 | | x ≥ 4.29 |
| Commercial Trade | Mammals and birds | units/month | 0 | 0 < x < 0.63 | 0.63 ≤ x < 1.80 | 1.8 ≤ x < 6 | | x ≥ 6 |
|  | Turtles and tortoises | units/month | 0 | 0 < x < 0.57 | 0.57 ≤ x < 1.20 | 1.2 ≤ x < 3.9 | | x ≥ 3.9 |

*Results are presented merging categories 4 and 5, for clarity.


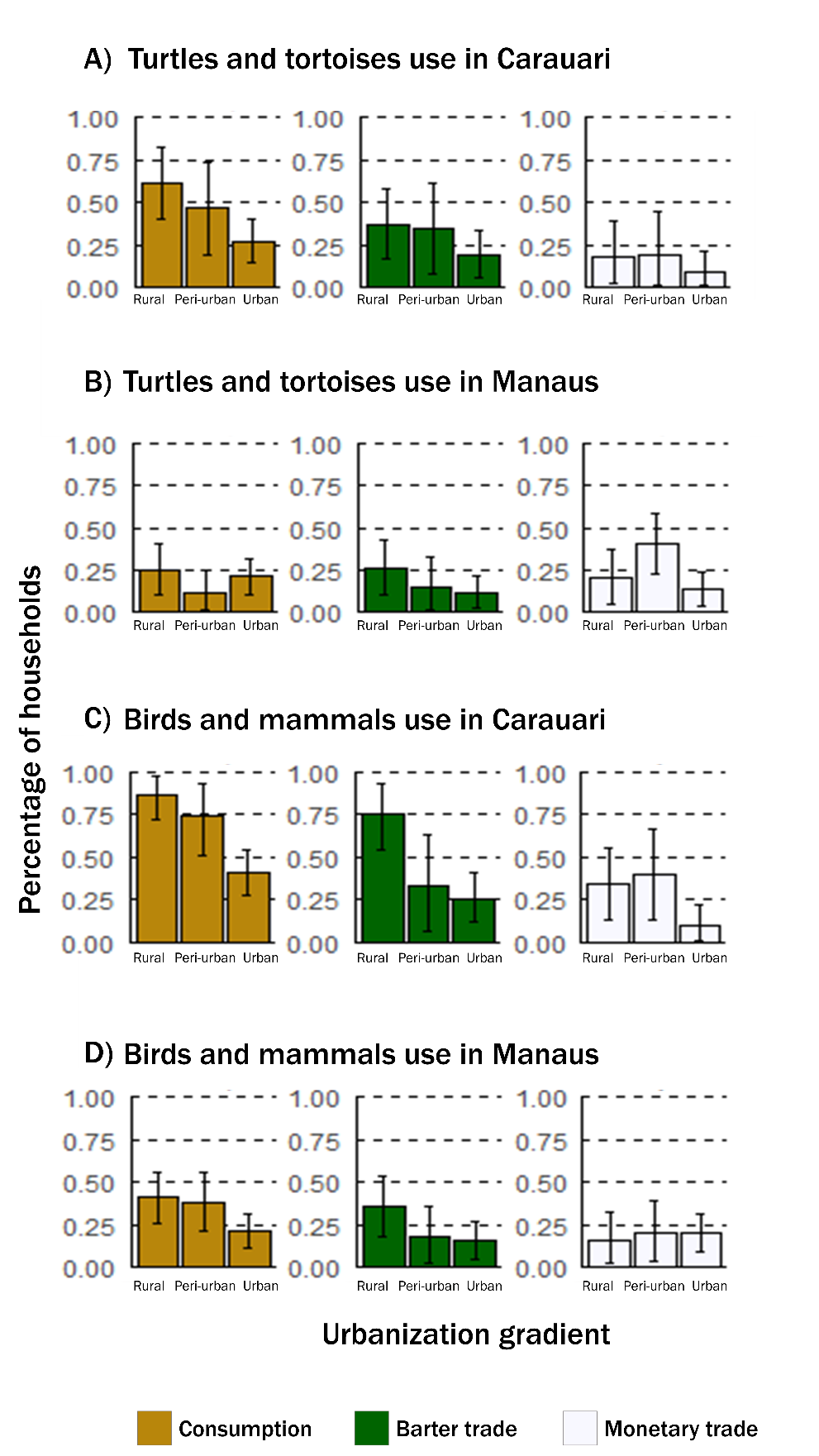


**Appendix S6 –**  Percentage of households that used: turtles and tortoises in Carauari, small town (A), and in Manaus, large city (B); birds and mammal meat in Carauari (C) and in Manaus (D) across the gradient of urbanization during the wet season (between december 2021 and may of 2022). Error bars are 95% credible intervals.


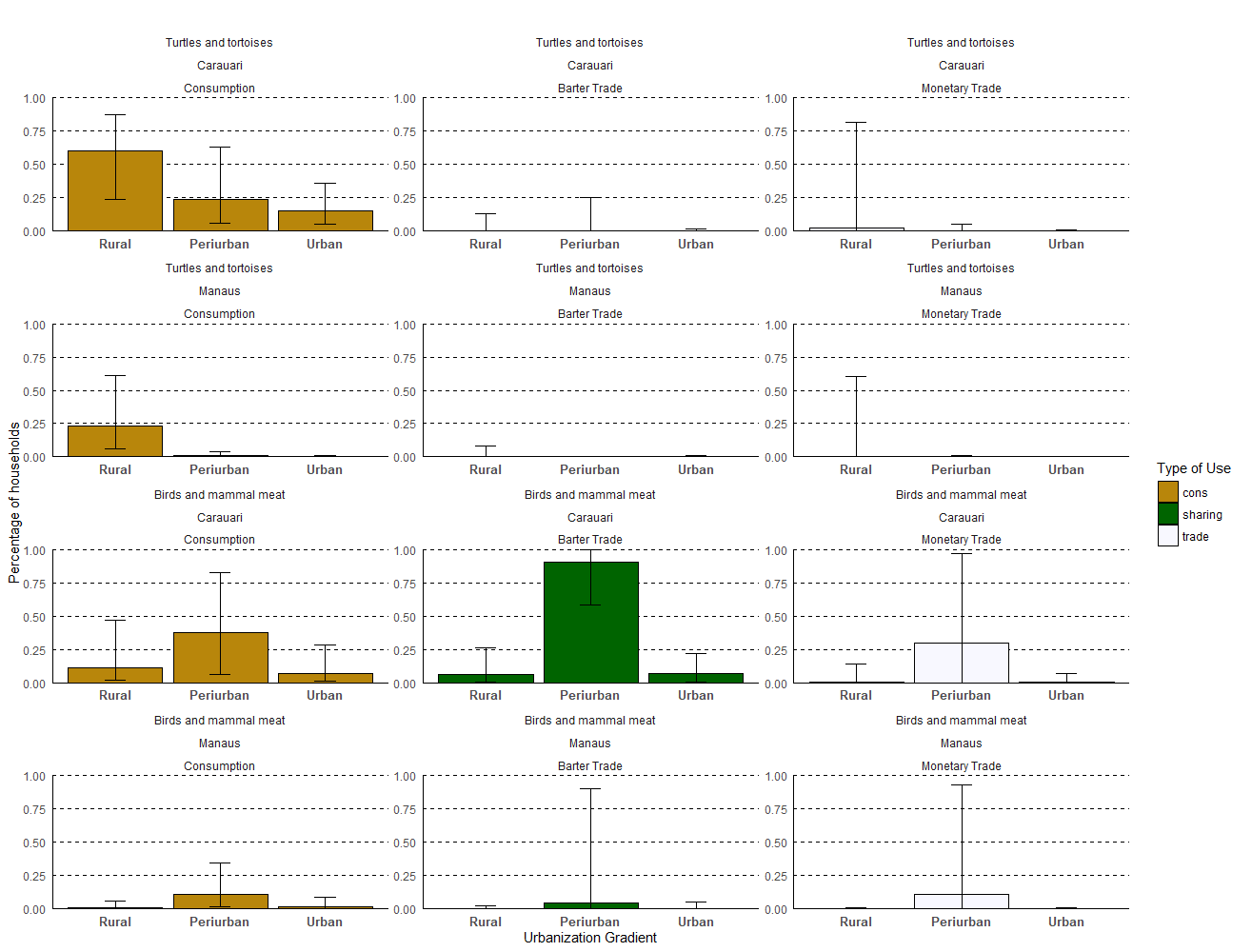


**Appendix S7 –**  Percentage of households that: consumed turtle and tortoise’ meat at a frequency of at least 1.11 animals/month; bartered turtle and tortoise meat at a frequency of at least 3 animals/month; traded turtle and tortoise meat at a frequency of at least 1.2 animals/month in Carauari, small town (A) and in Manaus, large city (B); and that: consumed birds and mammal meat at a frequency of at least 4.03 kg/month; bartered birds and mammal meat at a frequency of at least 1.8 kg/month; traded mammals and birds at a frequency of at least 1.8 animals/month in Carauari (C) and in Manaus (D) across the gradient of urbanization during the dry season (between June-November) of 2021. Error bars are 95% credible intervals.


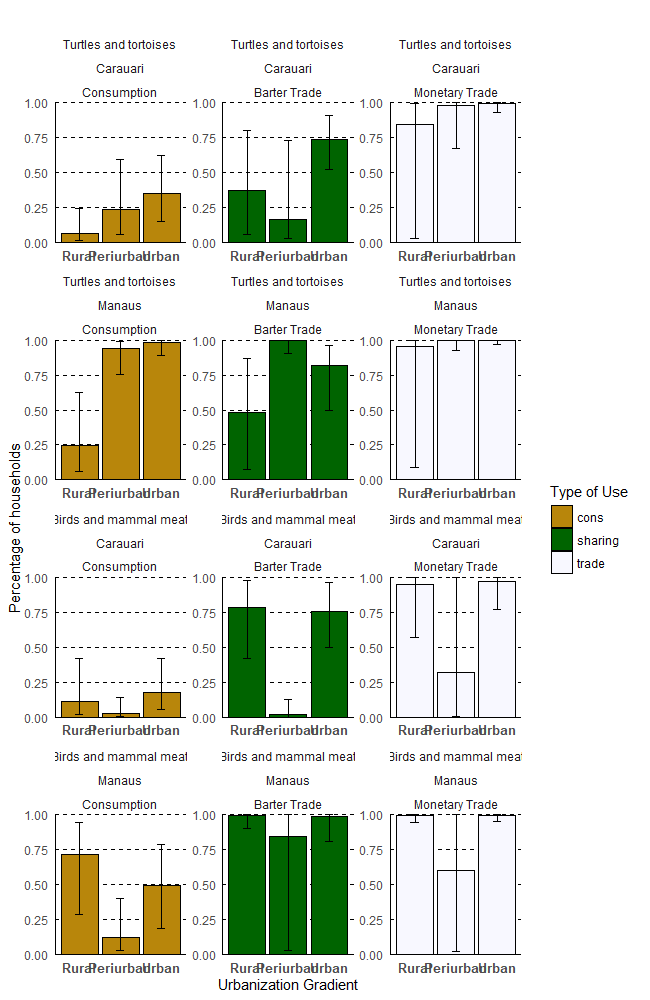


**Appendix S8 –**  Percentage of households that did not consumed turtle and tortoise’ meat; bartered turtle and tortoise meat; traded turtle and tortoise meat in Carauari, small town (A) and in Manaus, large city (B); and that did not: consumed birds and mammal meat; bartered birds and mammal meat; traded mammals and birds in Carauari (C) and in Manaus (D) across the gradient of urbanization during the dry season (between June-November) of 2021. Error bars are 95% credible intervals


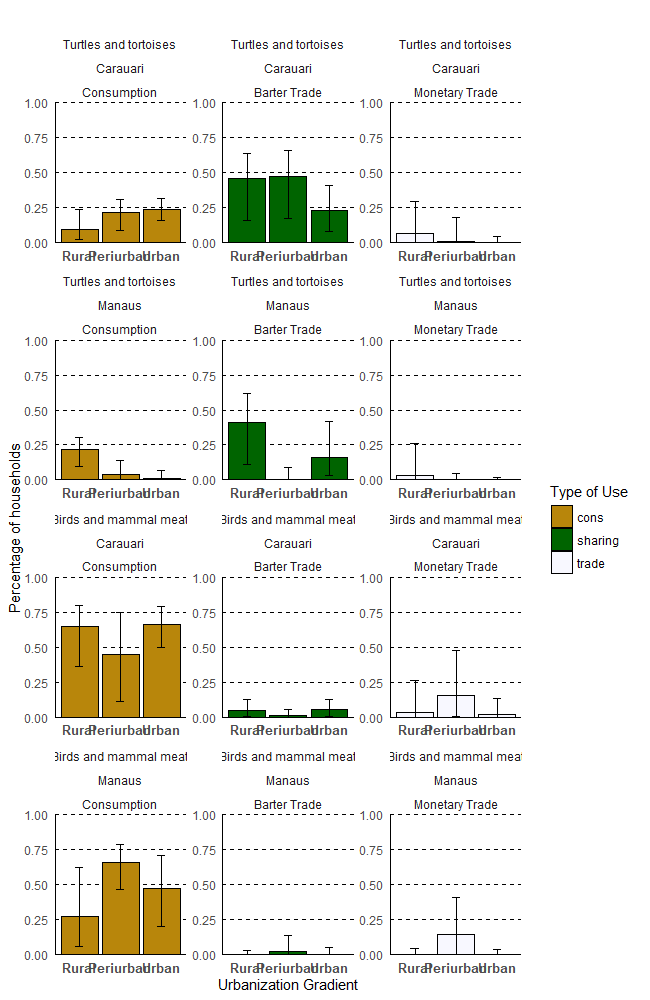


**Appendix S9 –**  Percentage of households that: consumed turtle and tortoise’ meat at a frequency of 0.36 animals/month; bartered turtle and tortoise meat at a frequency of at least 1.5 animals/month; traded turtle and tortoise meat at a frequency of at least 0.57 animals/month in Carauari, small town (A) and in Manaus, large city (B); and that: consumed birds and mammal meat at a frequency of 2.01 kg/month; bartered birds and mammal meat at a frequency of 0.42 kg/month; traded mammals and birds at a frequency of 0.63 animals/month in Carauari (C) and in Manaus (D) across the gradient of urbanization during the dry season (between June-November) of 2021. Error bars are 95% credible intervals


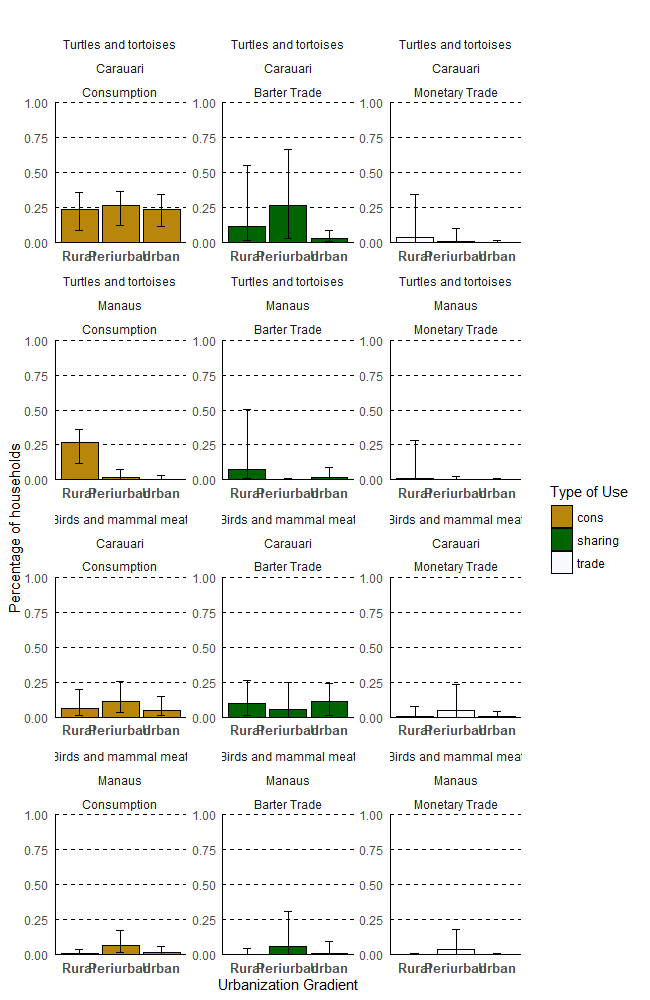


**Appendix S10 –**  Percentage of households that: consumed turtle and tortoise’ meat at a frequency of 0.36-1.11 animals/month; bartered turtle and tortoise meat at a frequency of 1.5-3 animals/month; traded turtle and tortoise meat at a frequency of 0.57-1.2 animals/month in Carauari, small town (A) and in Manaus, large city (B); and that: consumed birds and mammal meat at a frequency of 2.01-4.03 kg/month; bartered birds and mammal meat at a frequency of 0.42-1.8 kg/month; traded mammals and birds at a frequency of 0.63-1.8 animals/month in Carauari (C) and in Manaus (D) across the gradient of urbanization during the dry season (between June-November) of 2021. Error bars are 95% credible intervals


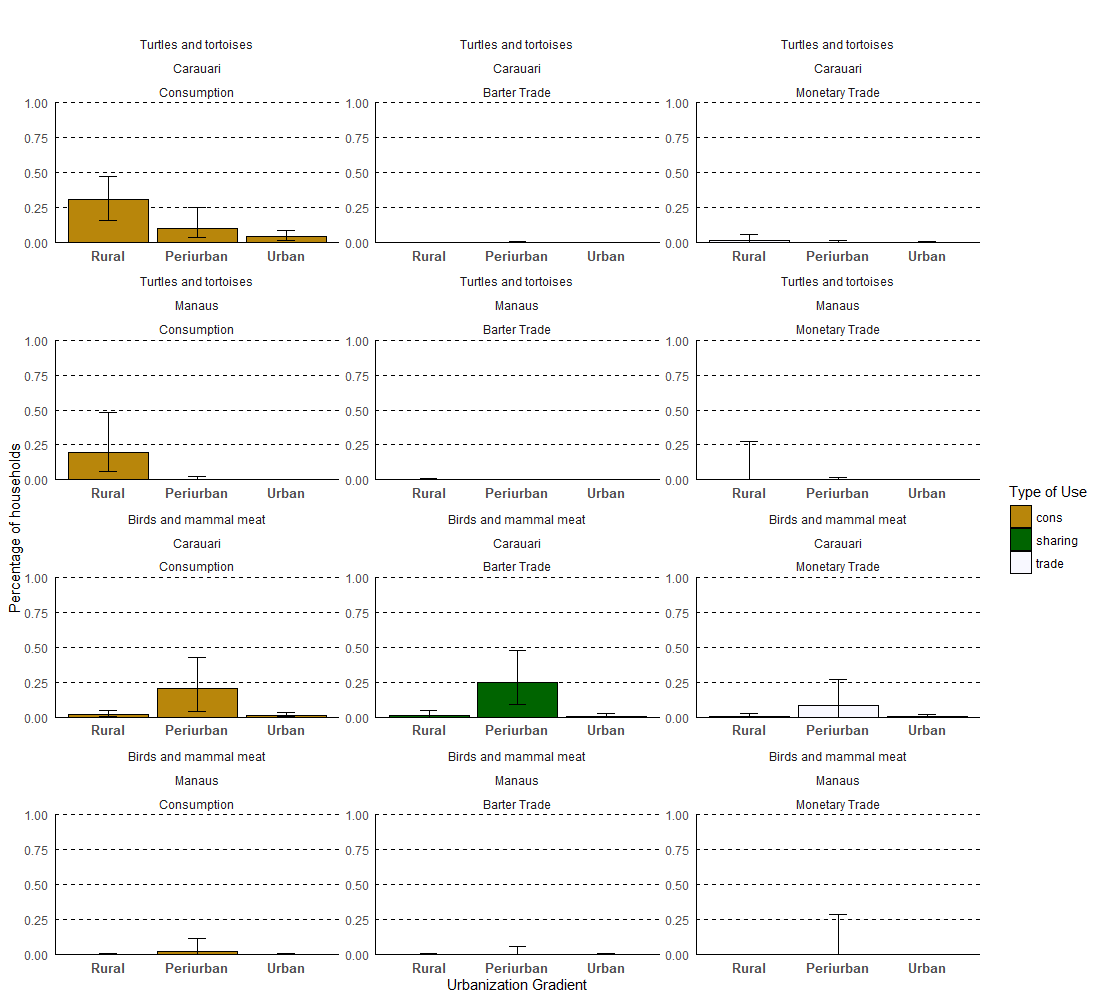


**Appendix S11 –**  Percentage of households that: consumed turtle and tortoise’ meat at a frequency of at least 1.11 animals/month; bartered turtle and tortoise meat at a frequency of at least 3 animals/month; traded turtle and tortoise meat at a frequency of at least 1.2 animals/month in Carauari, small town (A) and in Manaus, large city (B); and that: consumed birds and mammal meat at a frequency of at least 4.03 kg/month; bartered birds and mammal meat at a frequency of at least 1.8 kg/month; traded mammals and birds at a frequency of at least 1.8 animals/month in Carauari (C) and in Manaus (D) across the gradient of urbanization during the wet season (between December 2021 and May of 2022). Error bars are 95% credible intervals.


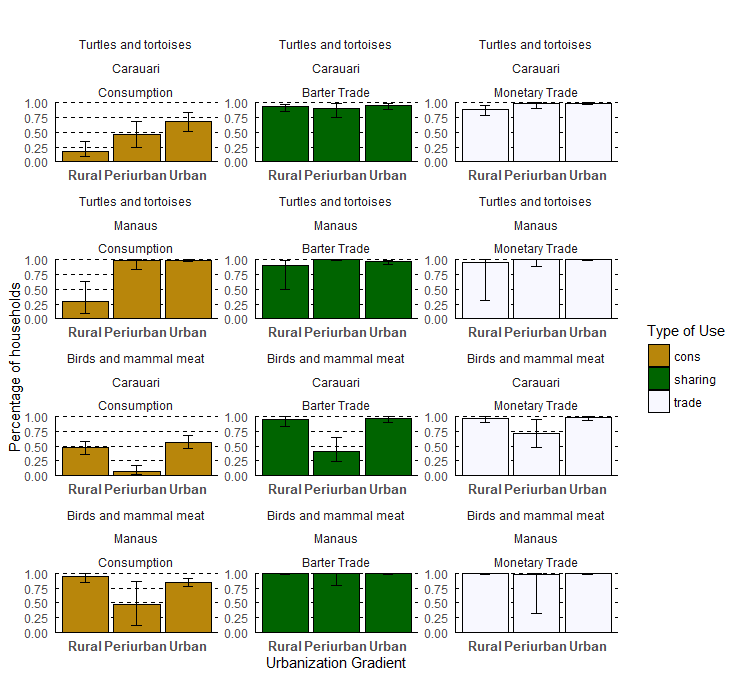


**Appendix S12 –**  Percentage of households that did not: consumed turtle and tortoise’ meat; bartered turtle and tortoise meat at a frequency; traded turtle and tortoise meat in Carauari, small town (A) and in Manaus, large city (B); and that did not: consumed birds and mammal meat; barterded birds and mammal meat; traded mammals and birds in Carauari (C) and in Manaus (D) across the gradient of urbanization during the wet season (between December 2021 and May of 2022). Error bars are 95% credible intervals.


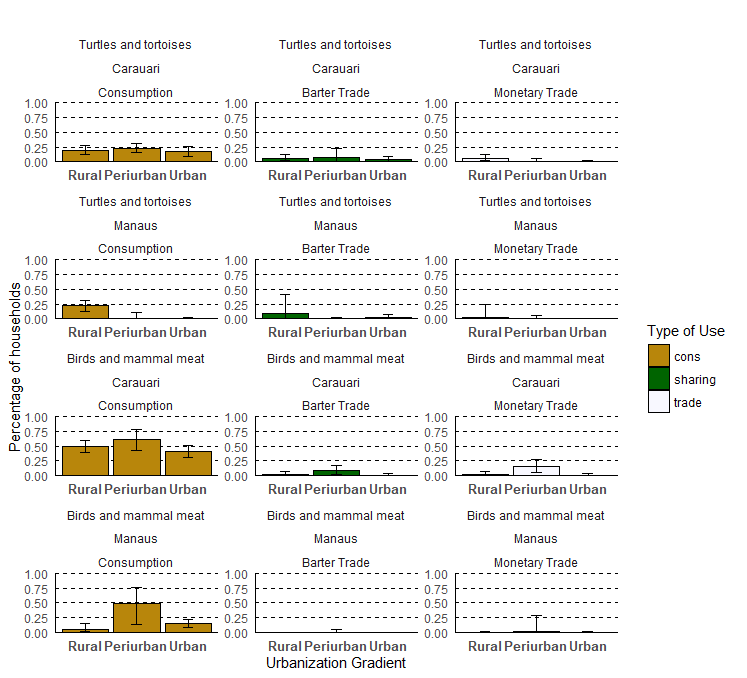


**Appendix S13 –**  Percentage of households that: consumed turtle and tortoise’ meat at a frequency of 0.36 animals/month; bartered turtle and tortoise meat at a frequency of at least 1.5 animals/month; traded turtle and tortoise meat at a frequency of at least 0.57 animals/month in Carauari, small town (A) and in Manaus, large city (B); and that: consumed birds and mammal meat at a frequency of 2.01 kg/month; bartered birds and mammal meat at a frequency of 0.42 kg/month; traded mammals and birds at a frequency of 0.63 animals/month in Carauari (C) and in Manaus (D) across the gradient of urbanization during the wet season (between December 2021 and May of 2022). Error bars are 95% credible intervals.


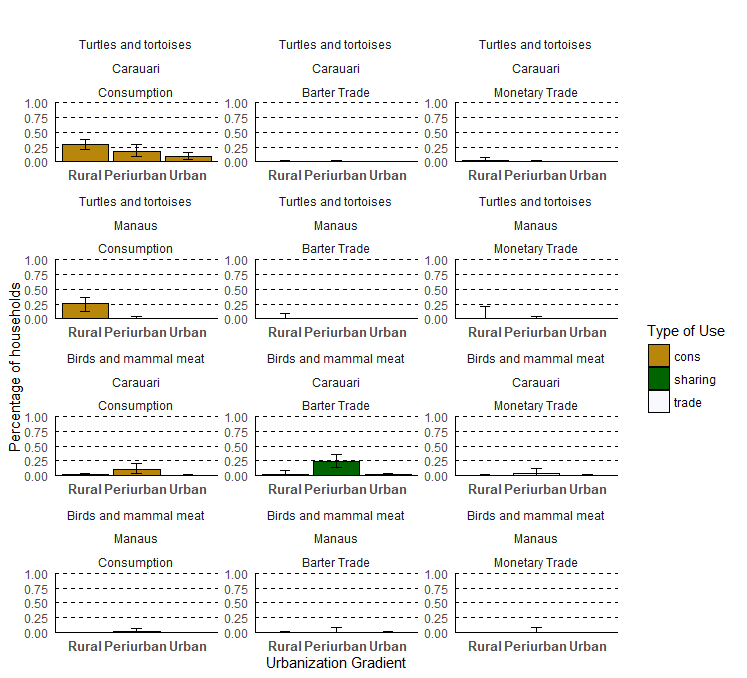


**Appendix S14 –**  Percentage of households that: consumed turtle and tortoise’ meat at a frequency of 0.36-1.11 animals/month; bartered turtle and tortoise meat at a frequency of 1.5-3 animals/month; traded turtle and tortoise meat at a frequency of 0.57-1.2 animals/month in Carauari, small town (A) and in Manaus, large city (B); and that: consumed birds and mammal meat at a frequency of 2.01-4.03 kg/month; bartered birds and mammal meat at a frequency of 0.42-1.8 kg/month; traded mammals and birds at a frequency of 0.63-1.8 animals/month in Carauari (C) and in Manaus (D) across the gradient of urbanization during the wet season (between December 2021 and May of 2022). Error bars are 95% credible intervals.

**Appendix S15 –**  Effects of urbanization and social factors on wildlife use. * Refers to significant effects of predictor variables. Variables were significant if their 95% credible interval did not overlap zero.

| **Model** | **Type of analysis** | **Response variable** | **Predictor variables**** | **Effect direction** | **Ratio** | **2.5% CI** | **97.5% CI** |
| --- | --- | --- | --- | --- | --- | --- | --- |
| Spatial factors associated with access to urban markets by rural and peri-urban households | Negative Binomial Regression | Number of visits to the urban center | Municipality* | +* | 1.3* | 1.03* | 1.64* |
|  |  |  | Multisited | + | 1.2 | 0.899 | 1.61 |
|  |  |  | Peri-urban* | +* | 3.02* | 2.42* | 3.77* |
| Spatial factors associated with access to rural resources by urban households | Negative Binomial Regression | Number of visits to the rural area | Municipality* | +* | 0.24* | 0.15* | 0.39* |
|  |  |  | Multisited* | +* | 4.78* | 2.79* | 8.18* |
| Spatial factors associated with social factors | Logistic Regression | Prevalence of dual-adult households | Municipality* | -* | 0.41 | 0.27* | 0.61* |
|  |  |  | Multisited | + | 1.60 | 0.99 | 2.59 |
|  |  |  | Peri-urban* | +* | 3.25* | 1.78* | 5.94* |
|  |  |  | Rural | +* | 1.60* | 1.06* | 2.43* |
|  | Logistic Regression | Prevalence of dependents (<18y.o.) over working age adults (≥18y.o.)*** | Municipality* | -* | 0.54* | 0.39* | 0.75* |
|  |  |  | Multisited | + | 1.11 | 0.76 | 1.65 |
|  |  |  | Peri-urban* | +* | 1.94* | 1.26* | 3.04* |
|  |  |  | Rural* | +* | 1.94* | 1.34* | 2.82* |
| Spatial factors associated with wildlife consumption | Ordinal logistic regression | Kg of mammals and birds' meat consumed per month | Municipality* | -* | 0.14* | 0.07* | 0.25* |
|  |  |  | Multisited* | +* | 2.51* | 1.38* | 4.54* |
|  |  |  | Peri-urban* | +* | 3.42* | 1.64* | 7.23* |
|  |  |  | Rural* | +* | 7.56* | 3.94* | 15.02* |
|  |  |  | Season* | +* | 1.55* | 1.05* | 2.34* |
|  |  | Units of turtles and tortoises consumed per month | Municipality* | -* | 0.042* | 0.02* | 0.09* |
|  |  |  | Multisited | + | 1.87 | 0.92 | 3.98 |
|  |  |  | Peri-urban* | +* | 6.64* | 2.82* | 16.73* |
|  |  |  | Rural* | +* | 13.04* | 5.87* | 30.27* |
|  |  |  | Season* | +* | 7.09* | 3.92* | 14.04* |
| Spatial factors associated with wildlife barter | Ordinal logistic regression | Kg of mammals and birds' meat bartered per month | Municipality* | -* | 0.02* | 0.001* | 0.07* |
|  |  |  | Multisited* | -* | 0.14* | 0.03* | 0.47* |
|  |  |  | Peri-urban* | +* | 43.81* | 9.46* | 298.96* |
|  |  |  | Rural* | +* | 82.72* | 19.36* | 554.18* |
|  |  |  | Season | + | 1.5 | 0.71 | 3.13 |
|  |  | Units of turtles and tortoises bartered per month | Municipality* | -* | 0.24* | 0.08* | 0.54* |
|  |  |  | Multisited | + | 1.31 | 0.54 | 2.87 |
|  |  |  | Peri-urban* | +* | 5.77* | 2.26* | 15.96* |
|  |  |  | Rural* | +* | 5.12* | 2.02* | 12.95* |
|  |  |  | Season* | +* | 2.85* | 1.50* | 6.48* |
| Spatial factors associated with wildlife monetary trade | Ordinal logistic regression | Units of mammals and birds monetary traded per month | Municipality | - | 0.33 | 0.08 | 1.01 |
|  |  |  | Multisited | - | 0.74 | 0.21 | 2.42 |
|  |  |  | Peri-urban | + | 1.95 | 0.07 | 13.5 |
|  |  |  | Rural* | +* | 8.35* | 2.62* | 34.59* |
|  |  |  | Season* | +* | 2.48* | 1.02* | 6.95* |
|  |  | Units of turtles and tortoises monetary traded per month | Municipality* | -* | 0.30* | 0.11* | 0.72* |
|  |  |  | Multisited | - | 0.56 | 0.11 | 1.78 |
|  |  |  | Peri-urban | + | 2.28 | 0.71 | 7.17 |
|  |  |  | Rural | + | 2.25 | 0.79 | 6.71 |
|  |  |  | Season* | +* | 25.92* | 4.50* | 277.40* |
| Visits to the rural area and wildlife consumption | Ordinal logistic regression | Kg of mammals and birds' meat consumed per month | Number of visits to the rural area* | +* | 1.36* | 1.08* | 1.78* |
|  |  |  | Season | + | 1.34 | 0.82 | 2.24 |
|  |  | Units of turtles and tortoises consumed per month | Number of visits to the rural area* | +* | 2.94* | 1.69* | 4.83* |
|  |  |  | Season* | +* | 16.93* | 3.63* | 124.03* |
| Visits to the urban area and wildlife consumption | Ordinal logistic regression | Kg of mammals and birds' meat consumed per month | Number of visits to the urban center | - | 0.53 | 0.19 | 1.19 |
|  |  |  | Season | + | 1.41 | 0.81 | 2.45 |
|  |  | Units of turtles and tortoises consumed per month | Number of visits to the urban center | - | 0.91 | 0.28 | 1.59 |
|  |  |  | Season* | +* | 4.19* | 2.42* | 7.34* |
| Social factors associated with wildlife consumption | Ordinal logistic regression | Kg of mammals and birds' meat consumed per month | Dual-adult | + | 1.59 | 0.88 | 3.5 |
|  |  |  | Level of dependents | + | 1.15 | 0.91 | 1.45 |
|  |  |  | Season* | + | 1.36 | 0.95 | 1.94 |
|  |  | Units of turtles and tortoises consumed per month | Dual-adult | + | 1.15 | 0.54 | 2.84 |
|  |  |  | Level of dependents* | +* | 1.43* | 1.08* | 1.97* |
|  |  |  | Season* | +* | 2.95* | 1.89* | 4.71* |
| Visits to the rural area and wildlife barter | Ordinal logistic regression | Kg of mammals and birds' meat barter per month | Number of visits to the rural area | + | 1.64 | 0.76 | 2.89 |
|  |  |  | Season | + | 5.07 | 0.52 | 84.68 |
|  |  | Units of turtles and tortoises bartered per month | Number of visits to the rural area | - | 0.99 | 0.58 | 1.41 |
|  |  |  | Season | + | 1.29 | 0.39 | 4.4 |
| Visits to the urban area and wildlife barter | Ordinal logistic regression | Kg of mammals and birds' meat bartered per month | Number of visits to the urban center* | +* | 4.13* | 1.11* | 17.91* |
|  |  |  | Season | + | 1.4 | 0.77 | 2.65 |
|  |  | Units of turtles and tortoises bartered per month | Number of visits to the urban center | + | 1.3 | 0.76 | 2.19 |
|  |  |  | Season* | +* | 2.63* | 1.36* | 5.32* |
| Social factors associated with wildlife barter | Ordinal logistic regression | Kg of mammals and birds' meat bartered per month | Dual-adult* | +* | 10.13* | 1.41* | 167.63* |
|  |  |  | Level of dependents | + | 1.34 | 0.78 | 2.01 |
|  |  |  | Season | + | 2.35 | 0.99 | 7.57 |
|  |  | Units of turtles and tortoises bartered per month | Dual-adult | + | 2.47 | 0.88 | 8.34 |
|  |  |  | Level of dependents* | +* | 1.53* | 1.12* | 2.05* |
|  |  |  | Season* | +* | 2.51* | 1.31* | 5.44* |
| Visits to the rural area and wildlife monetary trade | Ordinal logistic regression | Units of mammals and birds' monetary traded per month | Number of visits to the rural area | + | 1.28 | 0.05 | 3.06 |
|  |  |  | Season | - | 0.62 | 0.001 | 21.97 |
|  |  | Units of turtles and tortoises monetary traded per month | Number of visits to the rural area | - | 0.95 | 0.19 | 1.52 |
|  |  |  | Season* | +* | 15.64* | 1.72* | 200.87* |
| Visits to the urban area and wildlife monetary trade | Ordinal logistic regression | Units of mammals and birds' monetary traded per month | Number of visits to the urban center | - | 0.15 | 0.006 | 1.09 |
|  |  |  | Season | + | 1.76 | 0.78 | 4.35 |
|  |  | Units of turtles and tortoises monetary traded per month | Number of visits to the urban center* | +* | 2.39* | 1.04* | 6.55* |
|  |  |  | Season* | +* | 12.76* | 2.07* | 176.69* |
| Social factors associated with wildlife monetary trade | Ordinal logistic regression | Units of mammals and birds' monetary traded per month | Dual-adult | + | 2.64 | 0.71 | 15.33 |
|  |  |  | Level of dependents* | +* | 1.52* | 1.06* | 2.12* |
|  |  |  | Season* | +* | 2.50* | 1.07* | 8.20* |
|  |  | Units of turtles and tortoises monetary traded per month | Dual-adult | - | 0.77 | 0.3 | 2.33 |
|  |  |  | Level of dependents | + | 1.25 | 0.86 | 1.75 |
|  |  |  | Season* | +* | 19.31* | 4.25* | 186.60* |

**Baseline variables were: for “Municipality” = Carauari, for “Multisited” = “Single-sited”, for “Peri-urban” and “Rural” = Urban, for “Season” = wet season, and for “Dual-adult” = Single-adult.
****We considered households with a greater number of people younger than 18y.o. than those of 18y.o. and older = 1, and greater number of people of 18.y.o. and older than those younger than 18y.o. = 0
